# Large rectangular cross-section tunnel undercrossing urban road by micro pipe jacking and joint assembly structure (MPJ & JAS) method in soft soils

**DOI:** 10.1038/s41598-024-55754-7

**Published:** 2024-03-01

**Authors:** Jia Zou, Xiongyao Xie, Biao Zhou, Chunzhao Jiang, Zhen Zhang, Jianjun Han, Qing Dai, Isam Shahrour

**Affiliations:** 1 Department of Geotechnical Engineering, College of Civil Engineering, Tongji University, Shanghai, 200092 China; 2 Shanghai Urban Construction Municipal Engineering (Group) Co., Ltd., Shanghai, 200092 China; 3 Shanghai Research Institute of Building Sciences Co., Ltd., Shanghai, 200092 China; 4 Shanghai Architectural and Engineering Consultants Co., Ltd., Shanghai, 200092 China; 5 Laboratoire de Genie Civil et Go-Environnement (LGCgE), Universit Lille1, 59650 Villeneuve dAscq, France

**Keywords:** Tunnel, MPJ & JAS method, CT-shaped integrated joint, Theoretical design method, Micro pipe jacking construction, Civil engineering, Mechanical engineering

## Abstract

With the continuous construction of urban traffic roads, more and more new roads are cut off by existing roads to form “dead end roads”. There is an urgent need for a trenchless method suitable for urban ultra-shallow overburden to build the undercrossing tunnel. To solve this problem, this paper proposed the micro pipe jacking and joint assembly structure (MPJ & JAS) method, which has the characteristics of shallow burial depth, low cost, short construction time, flexible cross-section setting and high space utilization. The MPJ & JAS method construct a large cross-section tunnel through assembling small cross-section elements, quite different from traditional methods. Therefore, this paper designed a CT-shaped integrated joint, the mechanical performance of which was verified and clarified by tensile test. The bending test and finite element (FE) analysis proved the reliability of MPJ & JAS tunnel structure, and confirmed the structure performances such as the failure models, crack behaviors, load–deflection response and stress–strain distribution. Moreover, the influences of the steel plate thickness, concrete strength and shear connector spacing were determined by the FE analysis. On the basis of test results and reasonable assumptions, a theoretical design method considering the influence of the CT-shaped integrated joint was proposed, which can effectively predict the bending strength of the MPJ & JAS tunnel structure with an error of less than 10%. Finally, in view of the characteristics of the MPJ & JAS method, the suitable micro pipe jacking machine, soil reinforcement measure, hydraulic traction construction technology, high-precision guidance system and concrete construction quality detection method based on the phased array ultrasonic imaging technology were developed, supporting the accurate and efficient construction of the MPJ & JAS tunnel.

## Introduction

With the rapid development of Chinese cities, their urban transportation system is facing increasing pressure. In order to reduce traffic pressure and facilitate urban travel, the skeleton road network composed of urban expressways and main roads has been formed. However, a large number of urban main roads are blocked by the urban expressway to form “dead end roads”^1^. It is necessary to use the trenchless technique to construct a tunnel undercrossing the existing road to open up the “dead end roads”.

As a trenchless technique, pipe roof method is widely adopted to construct a tunnel crossing roads, railways and airport runways in urban area. Especially, Japan has gradually developed and applied the pipe roof method, and invented some construction methods for passing through railways, such as the under railway road tunnelling (URT) method^2,3^, the prestressed concrete roof (PCR) method^4^, the high-speed element pull and jointed element structure (HEP & JES) method^5–7^, etc. Through the site instrumentation, experiment and theoretical analysis, it is found that pipe roof can reduce soil disturbance and ground settlement^8–10^. With the advantage of the settlement control of the pipe roof, the roof-box jacking (RBJ) method was formed by combining the pipe roof method and the box culvert jacking method^11,12^. Under the effect of the pipe curtain, the excavation face stability during the box culvert jacking process is improved, which significantly reduces the degree of environmental impact^13,14^. However, the RBJ method still has limitations such as long construction time and high cost because it involves pipe roof jacking and box culvert jacking. Moreover, in the case of construction in soft soil areas, in order to meet the requirements of settlement control, it is generally necessary to increase the cover depth of the tunnel. When the longitudinal gradient is constant, the transition distance between the ground road and tunnel entrance or exit will inevitably increase with the increase of the cover depth, resulting in increased construction costs and difficulties.

In order to reduce the construction time, cost and cover depth of tunnel, this paper proposed the micro pipe jacking and joint assembly structure (MPJ & JAS) method suitable for urban underpass and ultra-shallow overburden. Then, the reliability and structure performance of MPJ & JAS tunnel were verified and studied by the tensile test, bending test and finite element (FE) analysis, and a theoretical design method for MPJ & JAS tunnel structure was proposed. Finally, according to the characteristics of MPJ & JAS method, the corresponding construction equipment, technologies and detection method were developed.

## Outline of MPJ & JAS method

**Figure 1:**
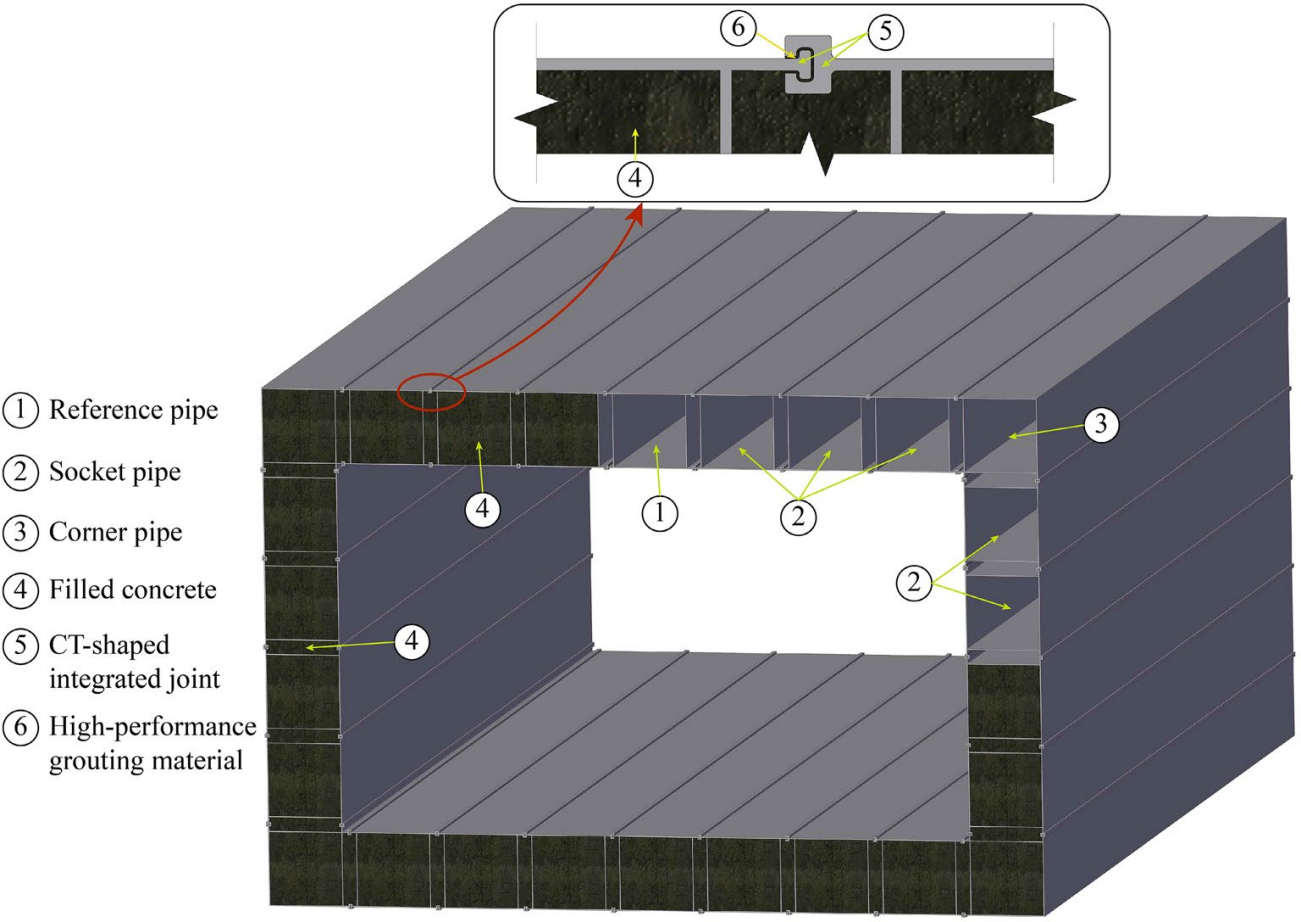
MPJ & JAS tunnel.

Whether the pipe roof method or RBJ method, the pipe roof is only used as the supporting structure during tunnel excavation or box culvert jacking construction. Pipe roof cannot be part of the tunnel lining, cannot be recycled after construction, and are of little use during tunnel operation. However, the MPJ & JAS method is quite different. Its core idea is to connect the small cross-section rectangular steel pipe element into a pipe roof through special joints, and then form a large cross-section box culvert structure through grouting in joint japs and filling concrete inside the pipe roof, as shown in Fig. 1. This structure is used as a supporting structure during tunnel excavation, and directly as a lining of the whole tunnel after excavation, which means that the pipe roof supporting structure is integrated with the tunnel lining.

### Construction method and process

**Figure 2:**
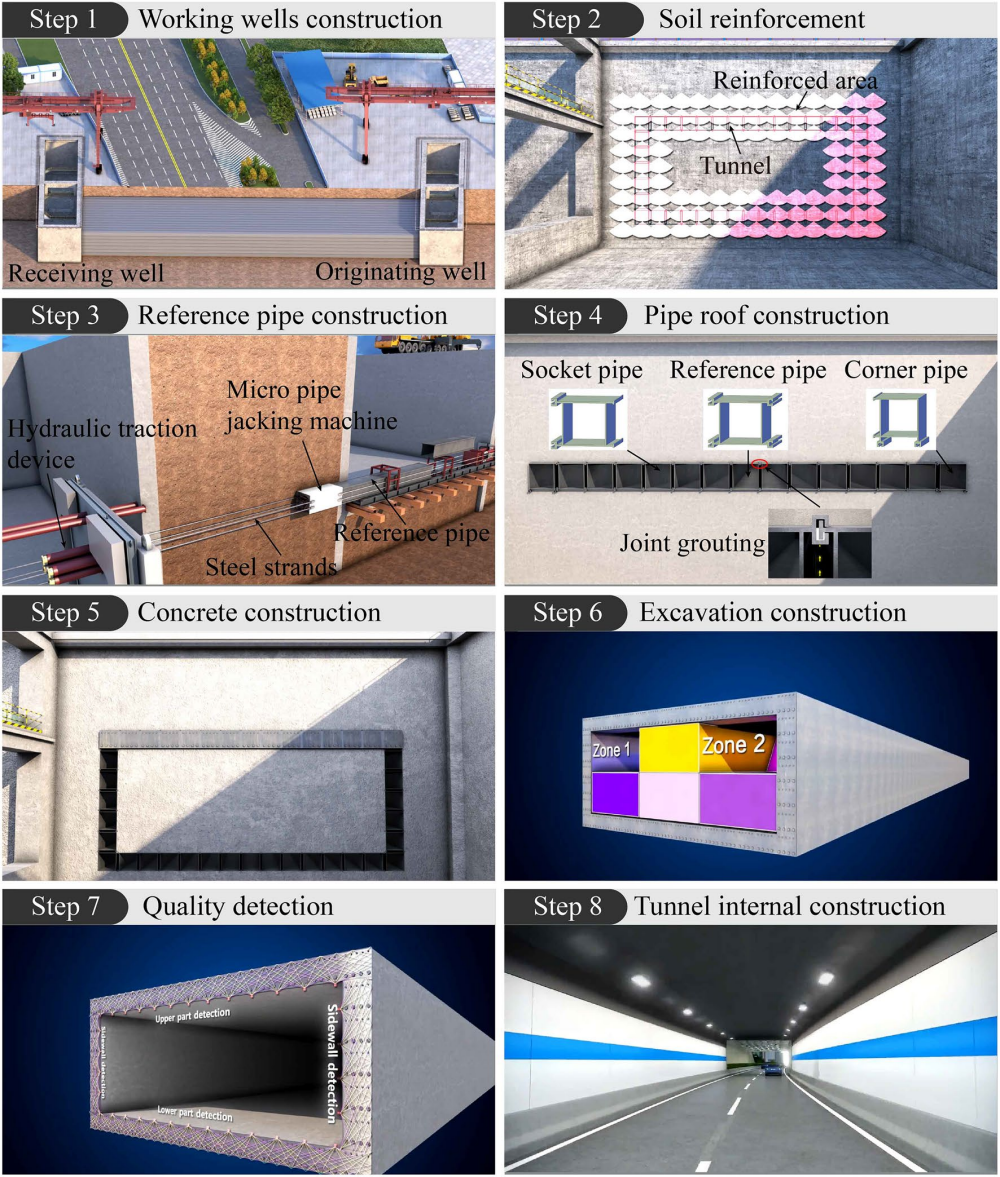
MPJ & JAS construction method and process.

Compared with pipe roof method and RBJ method, MPJ & JAS method has higher requirements for pipe roof construction and finer construction process. And different from the cast-in-situ tunnel lining after excavation or jacking box culvert structure, it forms a large cross-section tunnel lining by assembling small cross-section rectangular steel pipe elements. Therefore, the construction technique of MPJ & JAS method is quite special. The construction method and process (Fig. 2) is as follows in sequence:

1. Working wells construction: the originating well and receiving well are constructed by excavating foundation pits on both sides of the road to be crossed, and operation platforms and equipment are set up in wells.
2. Soil reinforcement: in the pipe roof jacking area, the special soil modifier is injected into the soil layer by horizontal grouting to reduce the disturbance of pipe jacking machine to the soil.
3. Reference pipe construction: in order to ensure the construction accuracy, the front traction method is used to construct the reference pipe. Steel strands used to pull the pipe jacking machine are installed by horizontal drilling, and then the hydraulic traction device is arranged in the receiving well to pull the rectangular micro pipe jacking machine and the reference pipe through four steel strands, so as to ensure the construction accuracy of the reference pipe.
4. Pipe roof construction: use the rectangular micro pipe jacking machine to jack the socket pipe connected with the reference pipe or the adjacent jacked socket pipe through the integrated joint. Until the turning point of the tunnel cross-section, the corner pipe is jacked to form the upper row of pipe roof. In the process of steel pipe jacking, the soil in the steel pipe is sent out synchronously by screw conveyor and conveyor belt. After the pipe roof is jacked, the gap of the integrated joint is grouted with high-performance grouting material.
5. Concrete construction: seal plates are welded at both ends of the upper row of pipe curtain, grouting holes are set at the lower part of the seal plate, and vent holes and grout overflow holes are set at the top. Then pour high-strength and self-compacting concrete into the pipe curtain to form the tunnel roof. Follow steps 4 and 5 to construct the tunnel side walls and the tunnel floor.
6. Excavation construction: excavate the soil inside the tunnel by zones manually or mechanically.
7. Quality detection: at the same time of excavation, the defects of concrete pouring inside the pipe roof are detected by phased array ultrasonic imaging technology, and the defects are filled by pilot hole grouting to ensure the quality of concrete pouring inside the pipe roof.
8. Tunnel internal construction: carry out internal waterproofing, pavement laying and internal decoration, and complete the tunnel construction.

### Main advantages

Generally, the construction of tunnel undercrossing the existing road in urban area inevitably faces challenges such as the shortage of construction land and underground space resources, the short transition distance between ground road and tunnel, and the control of construction time and cost. Therefore, the trenchless technology with small construction area, flexible and variable cross-section, high space utilization rate, short construction time, low cost and suitable for shallow overburden is needed to construct the tunnel undercrossing the existing road. MPJ & JAS method has significant advantages in these aspects, as shown in Table 1.

**Table 1:** Comparison of different methods for tunnel undercrossing urban road.

Method	Cover depth	Construction cost	Construction time	Cross-section layout	Space utilization	Construction land
Shield tunneling	0.8*D* ~ 1.0*D* or > 6 m^15^ (Deep)	High	Medium	Inflexible	Low	Big
RBJ	> 2.5*d* + 0.5 or ≥ 3 m^16,17^ (Medium)	Medium	Long	Inflexible	Low	Big
MPJ & JAS	< 2 m (Shallow)	Low	Short	Flexible	High	Small

^a^*D*: tunnel diameter.

^b^*d*: pipe-jacking diameter.

**Figure 3:**
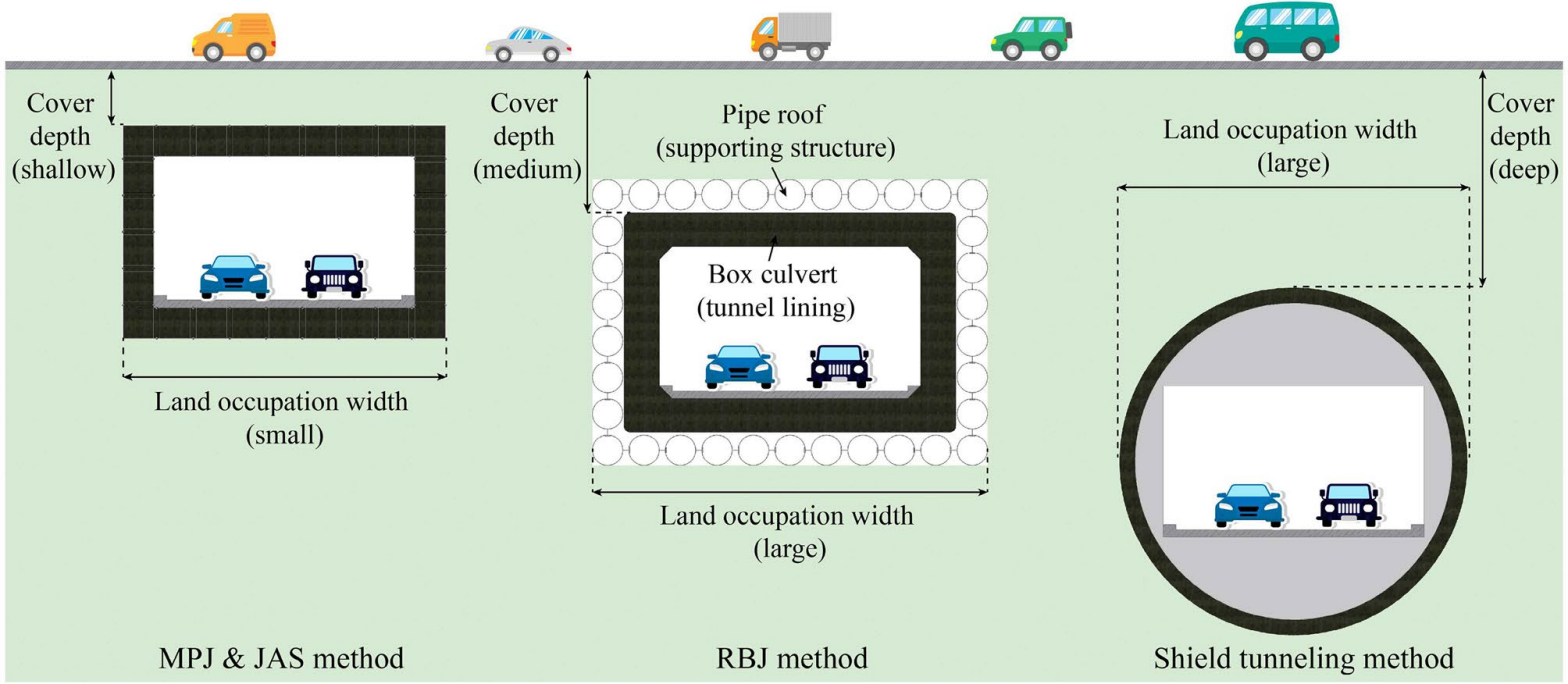
Comparison of MPJ & JAS method with RBJ method and shield tunneling method.

MPJ & JAS method is applicable to shallow overburden. Shield tunneling method involves the construction of large-diameter shield machine, which has a large disturbance to the soil, and requires a relatively thick overburden to control the ground settlement^18^. Even under the protection of pipe roof, RBJ method still needs to consider the influence of face stability on ground settlement during the jacking of large-section box culvert and soil excavation^19^. The MPJ & JAS method is to assemble a large cross-section tunnel through the small cross-section element, which means that the impact of the micro pipe jacking construction on the ground disturbance is very small. In addition, the tunnel lining has been formed before soil excavation, so the influence of face stability on ground settlement can be ignored. Therefore, MPJ & JAS method is more suitable for shallow overburden than traditional shield tunneling method and RBJ method, as shown in Fig. 3.

MPJ & JAS method has short construction time and low cost. Compared with RBJ method, MPJ & JAS method does not involve the jacking of large-section box culvert, which saves a lot of time and cost of box culvert construction.

MPJ & JAS method is flexible and variable in cross-section. It can easily adjust the tunnel cross-section size by increasing or reducing the number of small cross-section element, instead of redesigning the box culvert and its jacking scheme as in RBJ method, or replacing the shield machine of different sizes as in shield tunneling method.

MPJ & JAS method has high space utilization. On the one hand, its rectangular cross-section has a higher utilization rate of structural space than the circular cross-section constructed by the shield tunneling method. On the other hand, because it takes the steel pipe roof as a part of the tunnel lining, it saves the underground space occupied by the pipe roof outside the tunnel than the RBJ method.

MPJ & JAS method has small construction land. It does not need large-scale shield machine and box culvert, but only needs to meet the working space of micro pipe jacking machine, which means that working wells and construction land are relatively small.

## CT-shaped integrated joint

The design and performance of the joint is very important for connecting the discrete steel pipe elements into a whole tunnel lining.

### Design of joints and elements

**Figure 4:**
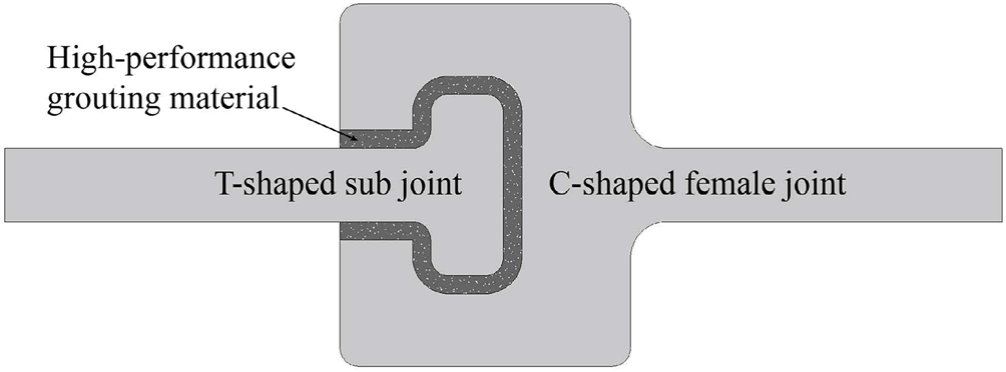
CT-shaped integrated joint.

MPJ & JAS method connects adjacent steel pipe elements with CT-shaped integrated joint (Fig. 4) composed of C-shaped female joint and T-shaped sub joint. High-performance grouting material is poured into the gap of the joint for force transmission. The CT-shaped integrated joint can bear the water and soil pressure during the pipe roof construction period and the tunnel operation period, and ensure the stable bearing capacity of MPJ & JAS tunnel.

**Figure 5:**
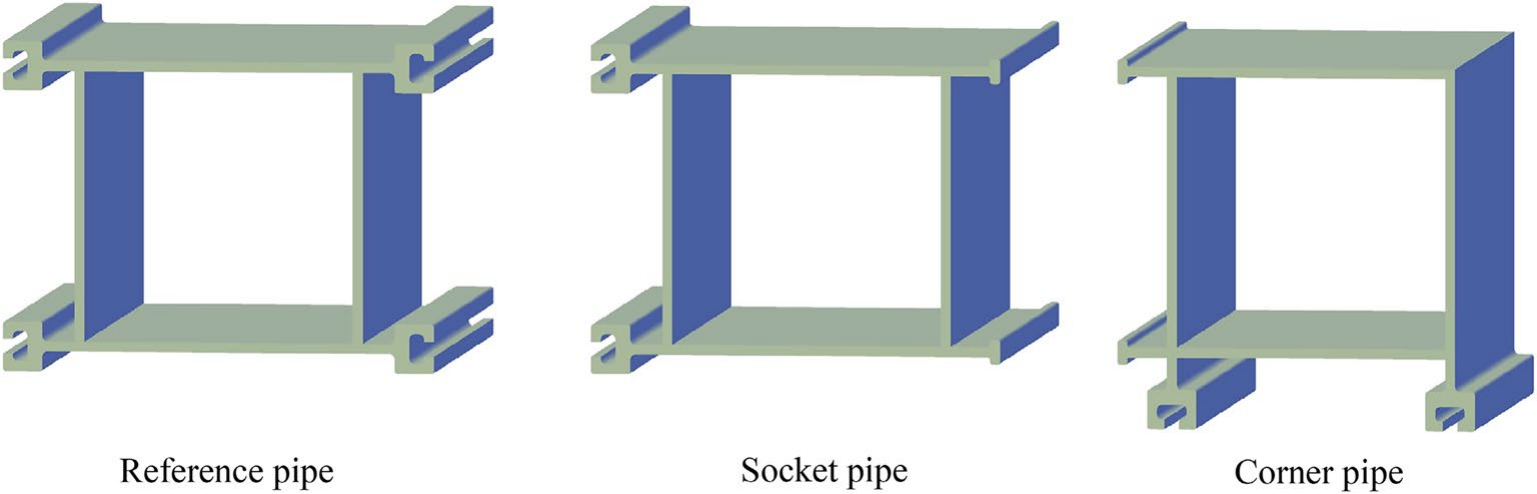
Design of steel pipe elements.

Based on the design of CT-type integrated joint, the steel pipe elements are divided into three categories as shown in Fig. 5. Reference pipe: the steel pipe element to be constructed first, which plays a guiding role in the subsequent steel pipe jacking, and the four corners are welded with C-shaped female joints; Socket pipe: the steel pipe element to be jacked through the guidance of the joint of the jacked steel pipe, with C-shaped female joint and T-shaped sub joint welded on the two opposite sides respectively; Corner pipe: the steel pipe element located at the corner of the cross-section of the tunnel, with C-shaped female joint and T-shaped sub joint welded on the two adjacent sides respectively, and used to realize the horizontal and vertical direction conversion.

### High-performance grouting material for joints

In order to improve the overall deformation resistance of the CT-shaped integrated joint, the grouting material filling the joint gap needs to meet certain construction and strength requirements:

1. The grouting material shall have high fluidity to prevent the formation of holes in the joints during construction.
2. The grouting material shall be free of bleeding and segregation to ensure the uniformity of the material and prevent the formation of connected holes.
3. The grouting material shall have slight expansion to make the joints fully filled.
4. The grouting material shall have high early and late strength to improve the overall bearing capacity of MPJ & JAS tunnel.

**Figure 6:**
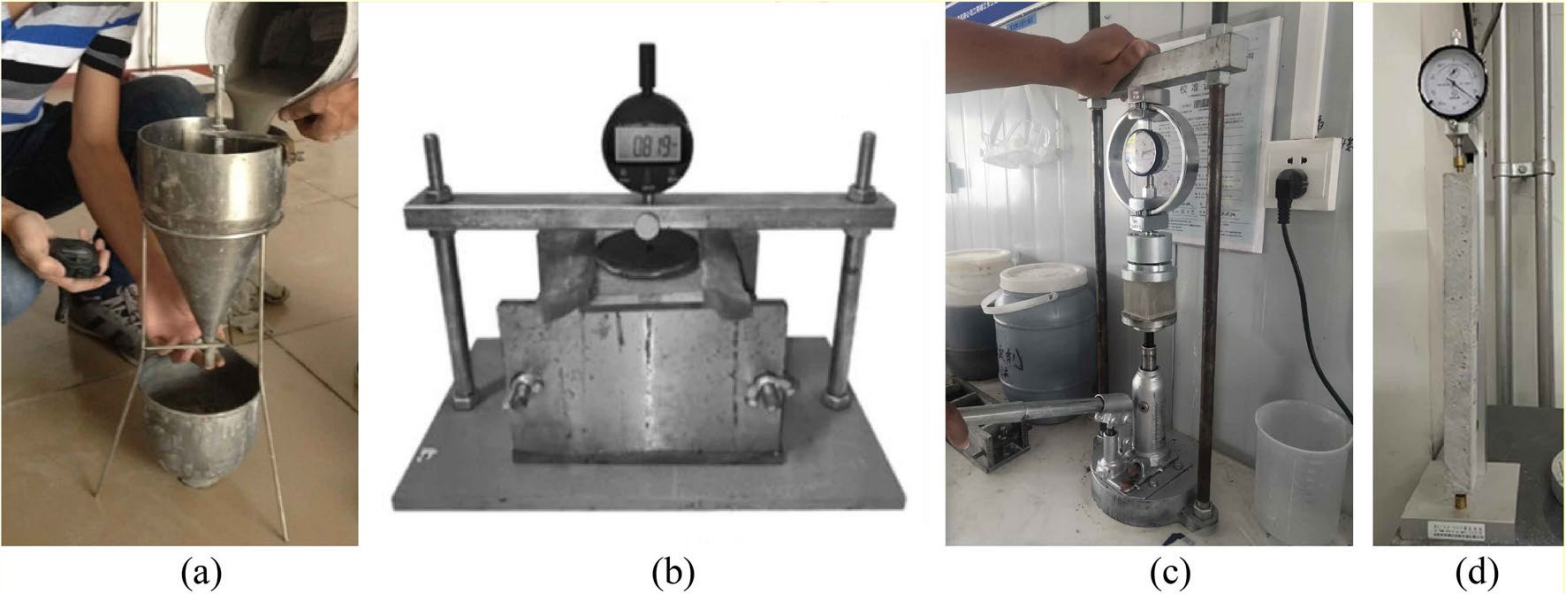
Grouting material test: (**a**) fluidity test, (**b**) expansion rate test, (**c**) compressive strength test, and (**d**) autogenous shrinkage test.

**Figure 7:**
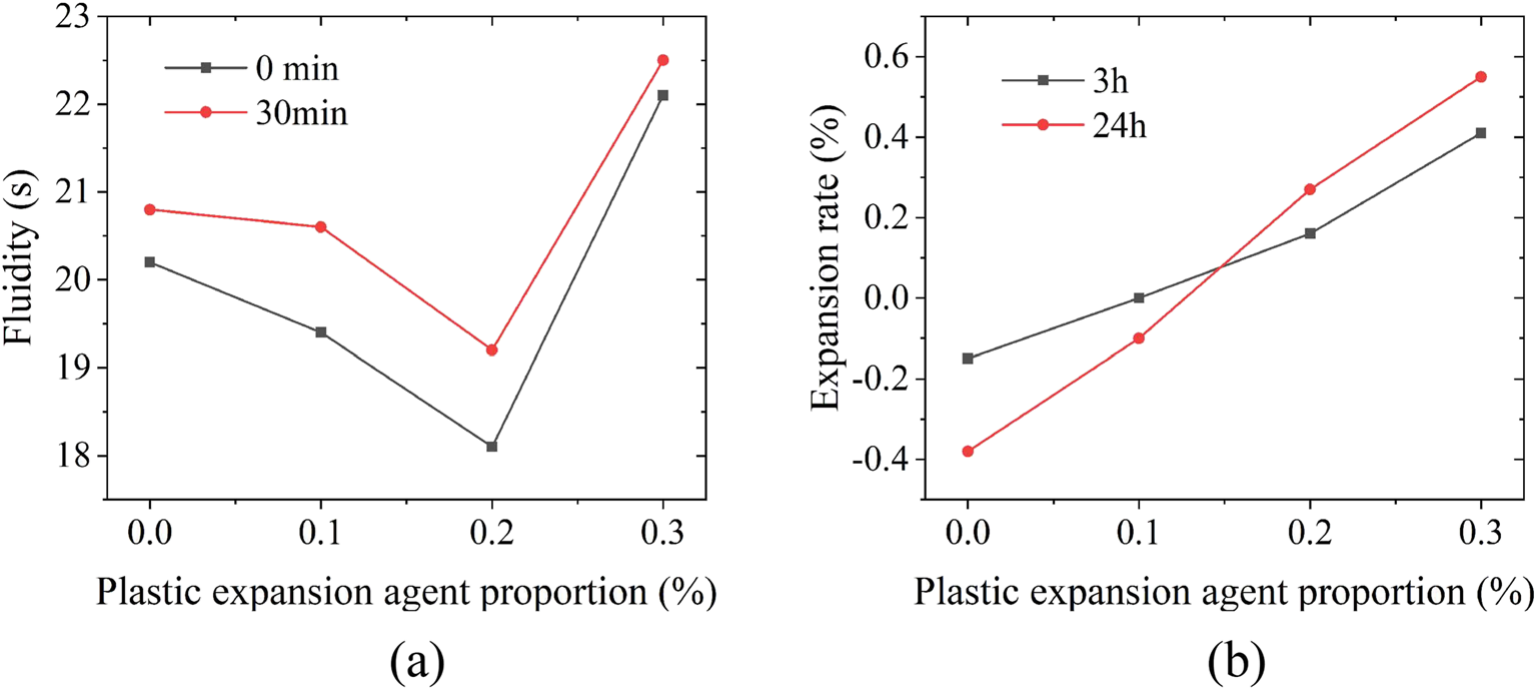
Test results of plastic expansion agent: (**a**) fluidity test, and (**b**) expansion rate test.

**Figure 8:**
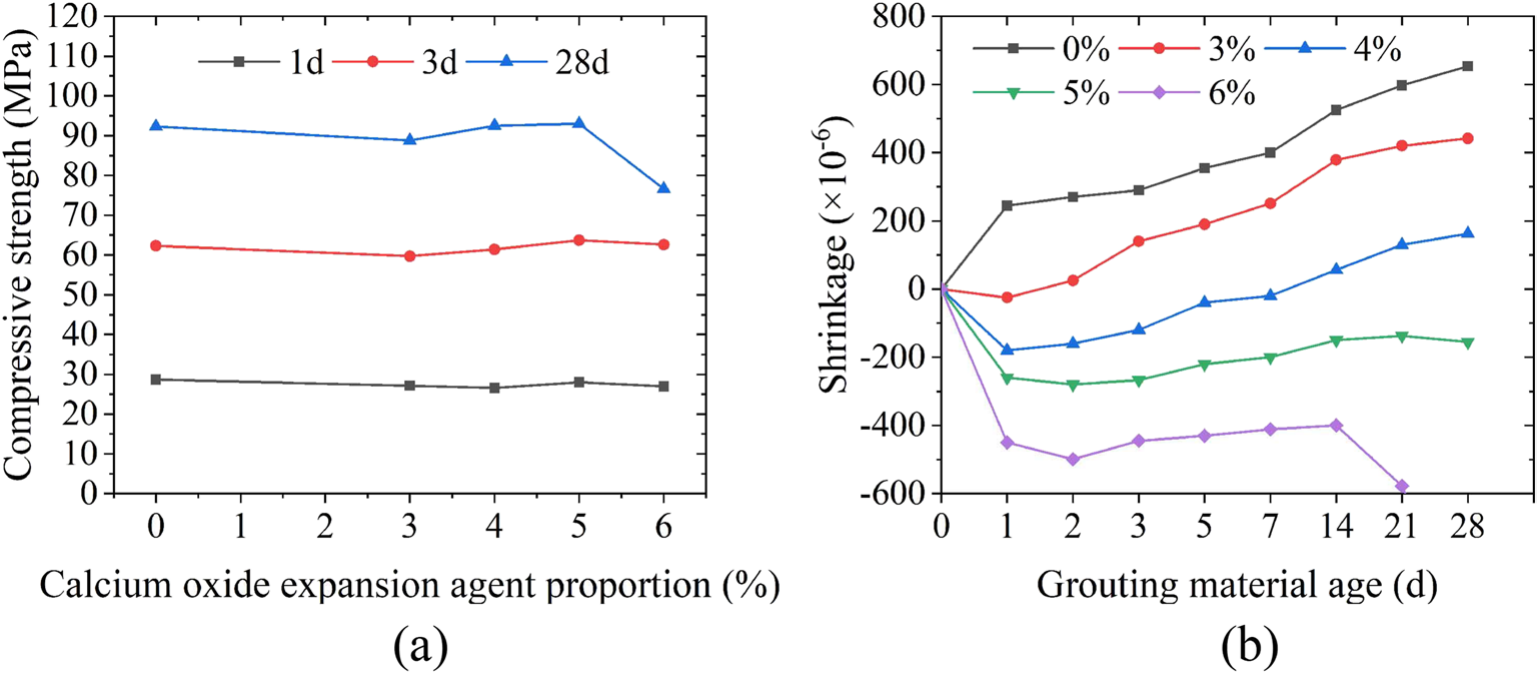
Test results of calcium oxide expansion agent: (**a**) compressive strength test, and (**b**) autogenous shrinkage test.

In order to prevent segregation and bleeding, the water-binder ratio is controlled at 0.28. With reference to the prestressed duct grouting material, according to the influence of water reducer, silica fume and fly ash on the fluidity, bleeding rate and compressive strength of grouting materials^20^, the mixing ratio is preliminarily determined to be 5% silica fume, 10% fly ash and 0.2% polycarboxylic acid water reducer to ensure the high fluidity of grouting materials. Aiming at the problem of large shrinkage of grouting materials, early plastic expansion agent and calcium oxide expansion agent are used to improve the strength and shrinkage performance of grouting materials^21^. Carry out fluidity test by the flow cone (see Fig. 6a), expansion rate test by the vertical expansion instrument (see Fig. 6b), compressive strength test by the uniaxial compression instrument (see Fig. 6c), and autogenous shrinkage test by the comparator (see Fig. 6d) in corresponding specifications to study the influence of plastic expansion agent and calcium oxide expansion agent on grouting materials^22–24^. Figure 7a shows that 0.2% plastic expansion agent meets the requirements of high fluidity, it overcomes the early shrinkage of grouting materials (see Fig. 7b); Fig. 8a shows that 5% calcium oxide expansive agent can ensure the compressive strength in the middle and later stage, and its shrinkage rate change curve is close to a stable state (see Fig. 8b), which means that the slurry volume is relatively stable and has good compensation ability for the shrinkage of the grouting material in the middle and later stage. Therefore, the optimal mix ratio of the high-performance grouting material is shown in Table 2.

**Table 2:** Optimal mix ratio of the high-performance grouting material.

Cement (%)	Silica fume (%)	Fly ash (%)	Polycarboxylic acid water reducer (%)	Plastic expansion agent (%)	Calcium oxide expansion agent (%)	Water-binder ratio
79.6	5	10	0.2	0.2	5	0.28

**Figure 9:**
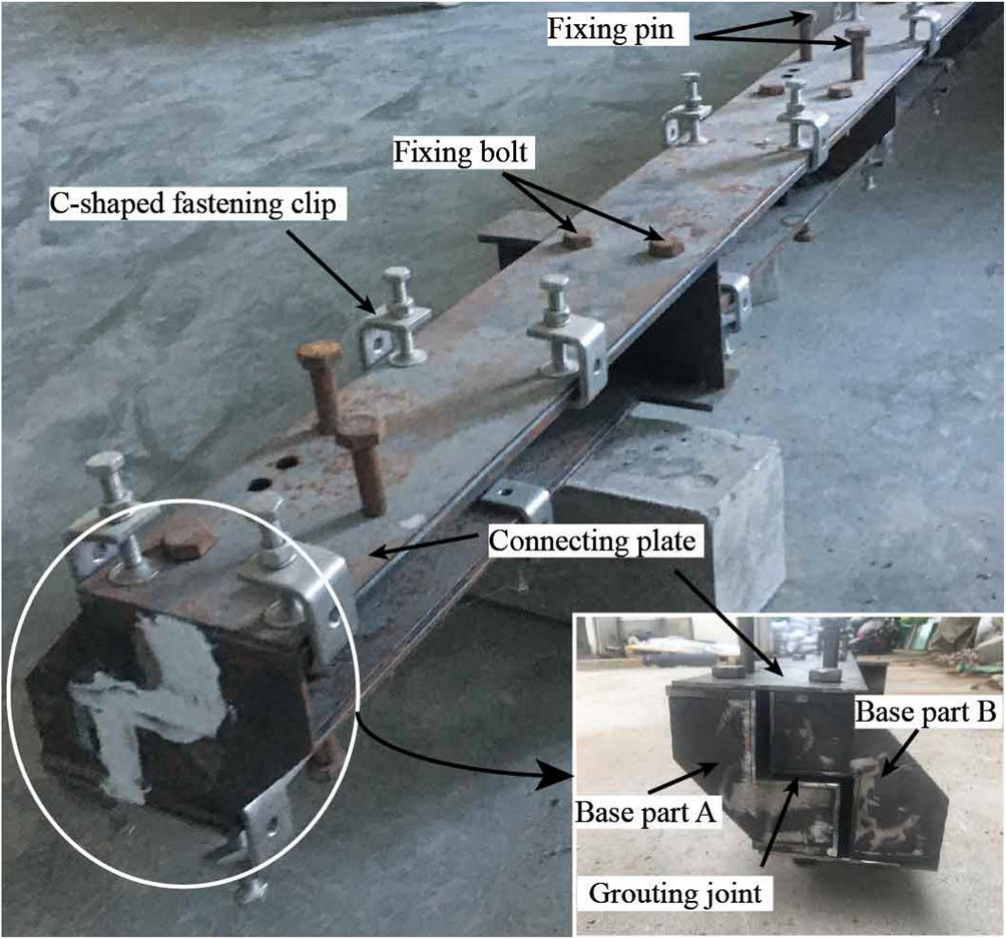
Indoor simulated grouting device.

**Figure 10:**
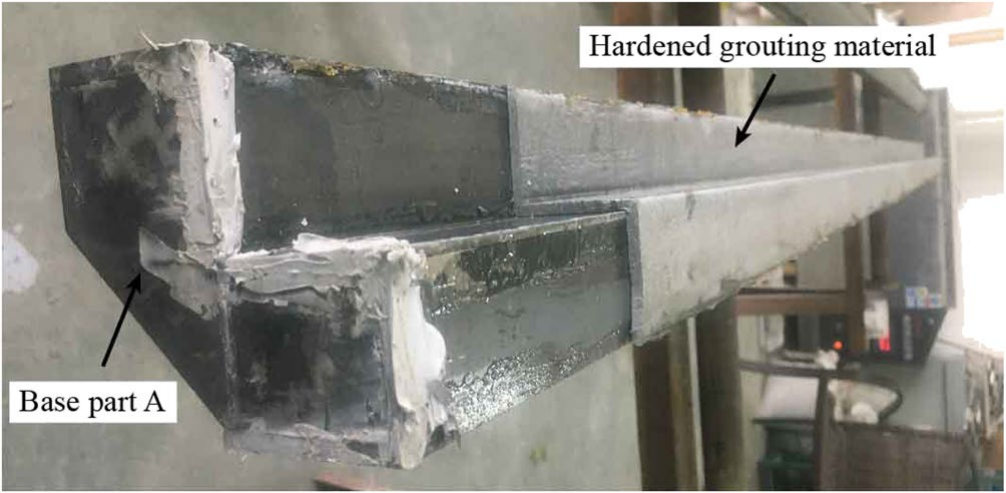
Simulated grouting test results.

In order to verify the feasibility of high-performance grouting materials in actual construction, the simulated grouting test was carried out on joints with gap widths of 3 mm, 4 mm, 5 mm, 7 mm, 9 mm, 11 mm (the CT-shaped integrated joint's gap width is 4 mm) through the indoor simulated grouting device (Fig. 9). Grout with a pressure of 0.5 MPa. After 28 days, remove the base steel parts on one side to observe the continuity of grouting materials and the defects such as pores and honeycombs. As shown in Fig. 10, the test results show that the high-performance grouting material has good filling and compaction effect for the joint, which proves that it can be used as the grouting material poured into the CT-shaped integrated joint in MPJ & JAS method.

### Joint tensile test

The tensile test for studying the tensile performance of the CT-shaped integrated joint were carried out to confirm the ability of connecting the steel pipe elements into a whole MPJ & JAS tunnel.

#### Experimental setup

**Figure 11:**
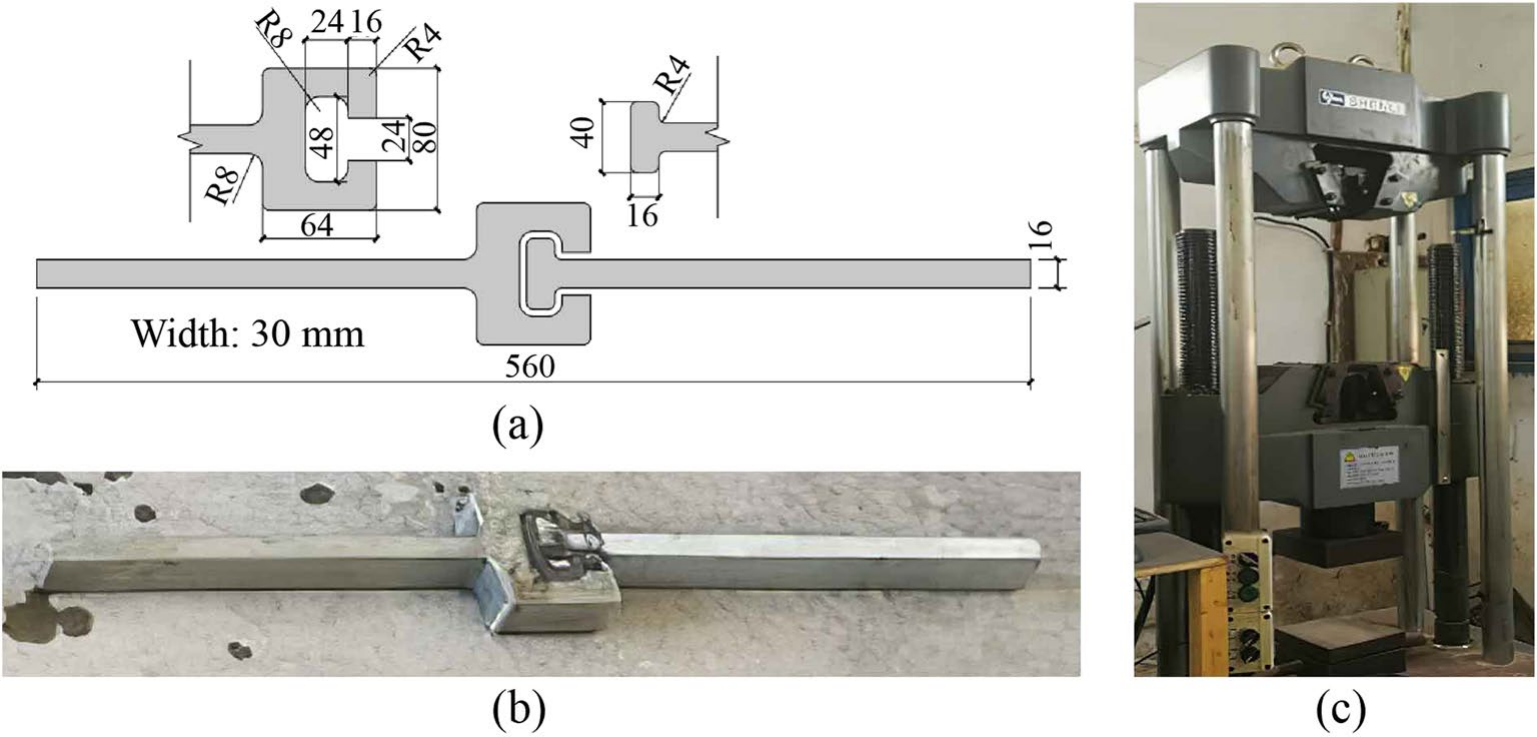
Tensile test specimen and machine.

As shown in Fig. 11, the CT-shaped integrated joint was 560 mm long, 30 mm wide, and 16 mm thick, its upper and lower ends were clamped by the multi-functional testing machine to stretch until the joint fails.

**Figure 12:**
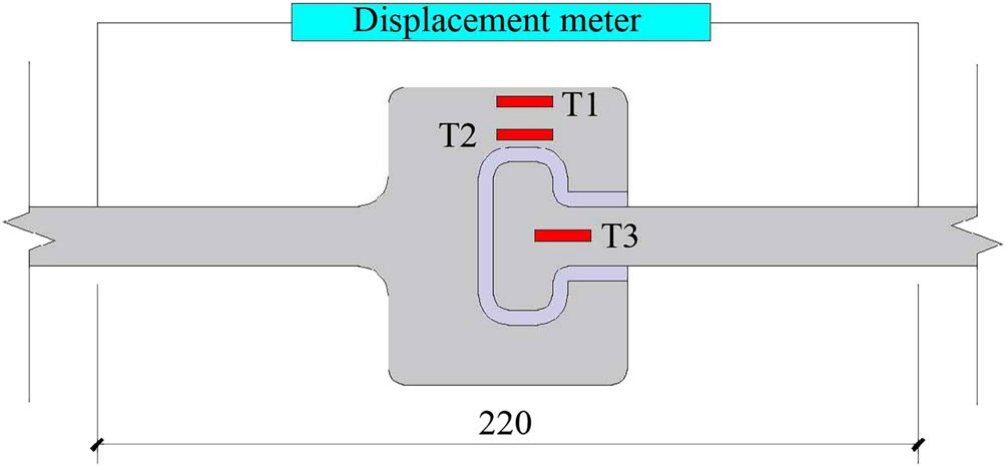
Measurement of the CT-shaped integrated joint.

Gauges were arranged on key parts of the CT-shaped integrated joint to measure the strain, as shown in Fig. 12. The axial tensile deformation of the joint was obtained by the displacement meter placed on both sides of the joint with a spacing of 220 mm. The tension force at both ends of the joint was obtained by the force sensor of the multi-functional testing machine.

#### Experimental results

**Figure 13:**
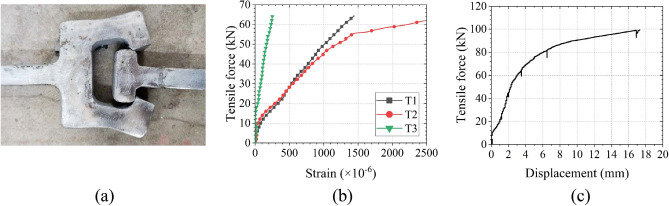
Tensile test results: (**a**) the failure shape, (**b**) the load–strain curves, and (**c**) the load–displacement curve.

During the tensile test, cracks first appeared at the edge of the grouting material. As tensile force increased, the grouting material in the joint gap was gradually crushed, and the joint was void. Finally, the joint was damaged due to excessive deformation of the interface, presenting a symmetrical failure shape in which the C-shaped female joint was opened, as shown in Fig. 13a. The deformation process of the joint is stable, maintaining a certain ductility and never breaking. The strain in the abdomen of the CT-shaped integrated joint was large, and it yielded when the tensile force reached 48 kN, as shown in Fig. 13b. These strain measurement results are consistent with the situation that the most severe deformation is in the abdomen of the CT-shaped integrated joint shown in Fig. 13a. The load–displacement curve in Fig. 13c indicates that the ultimate tensile strength of the CT-shaped integrated joint is quite high reaching 99.7 kN, and the axial deformation displacement at that value is 17.5 mm. The above test results prove that the mechanical properties of CT-shaped integrated joint are pretty good and can meet the bearing requirements of MPJ & JAS tunnel. When the joint reaches its ultimate tensile strength 99.7 kN and approaches a state of complete detachment, the ultimate tensile stress at both ends of the joint is 207.71 MPa (with a cross-sectional size of 30 mm wide and 16 mm thick), which serves as the failure criterion for the CT-shaped integrated joint.

## MPJ & JAS tunnel structure performance

Since MPJ & JAS tunnel is a novel and special elemental structure, it is necessary to carry out FE analysis and bending test to study and verify the structure performance as well as to determine the physical characteristics, failure behavior and strength for design.

### Bending test

#### Experimental design

**Figure 14:**
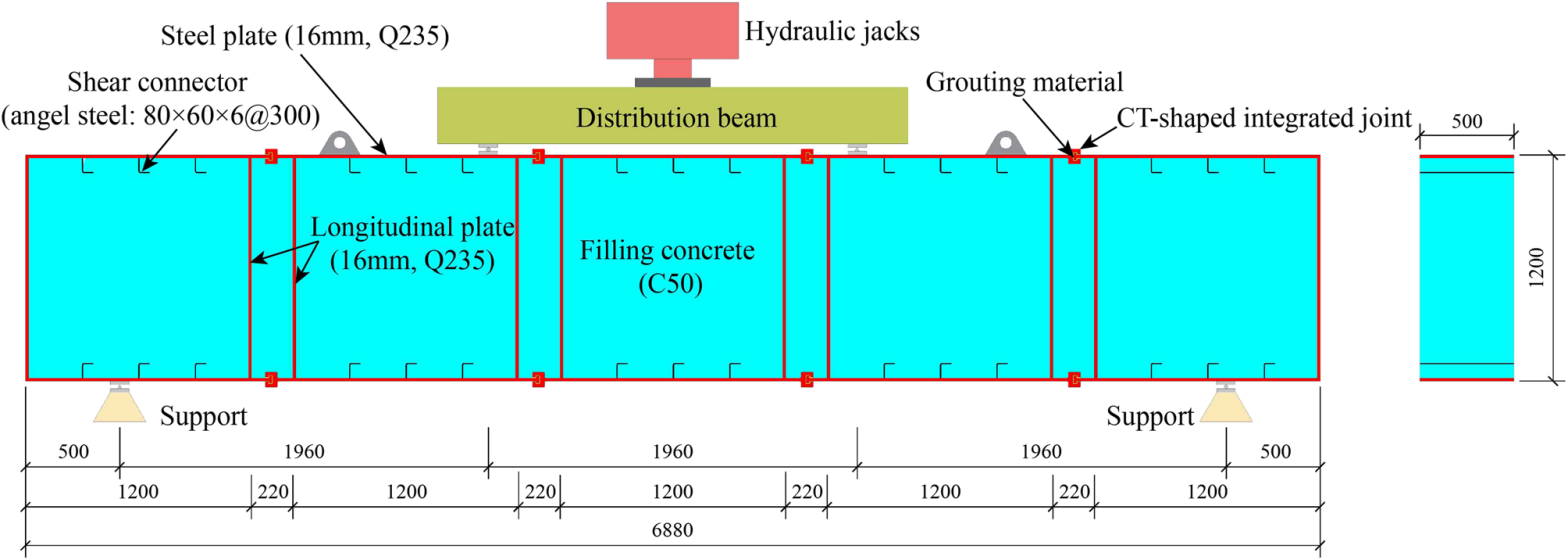
Arrangement of the bending test (in mm).

The specimen is a segment of MPJ & JAS tunnel structure composed of five steel pipe elements, as shown in Fig. 14. The main design parameters of the specimen are as follows: the steel plate thickness is 16 mm; the filled concrete strength grade in the specimen is C50; shear connectors with a spacing of 300 mm are arranged in the specimen as an optional constructional measure so the steel plate and the concrete cooperatively work; in addition, the steel plate and the shear connectors in the specimen are made of Q235 steel, and the high-performance grouting material (see in “High-performance grouting material for joints” section) is poured into the CT-shaped integrated joint.

The bending test specimen is supported by simple supports and is subjected to a four-point bending strength test. During the test, the load is applied by hydraulic jack and distribution beam. Before formally conducting the test, preloading is applied with loads of no more than 100 kN, with each load stage at 10 kN, and the load holding time is 5 min. Prior to structural cracking, each load stage is increased to 20 kN, and the load holding time is 5 min. From the onset of structural cracking until the cracks approach through penetration, each load stage is set at 100 kN, with a loading rate not exceeding 10 kN/s, and the load holding time is 5 min. From the point where the cracks approach through penetration to structural failure, the loading is controlled by displacement, with each stage at 4 mm, a loading rate not exceeding 0.5 mm/s, and the load holding time is 5 min.

#### Experimental measurements

**Figure 15:**
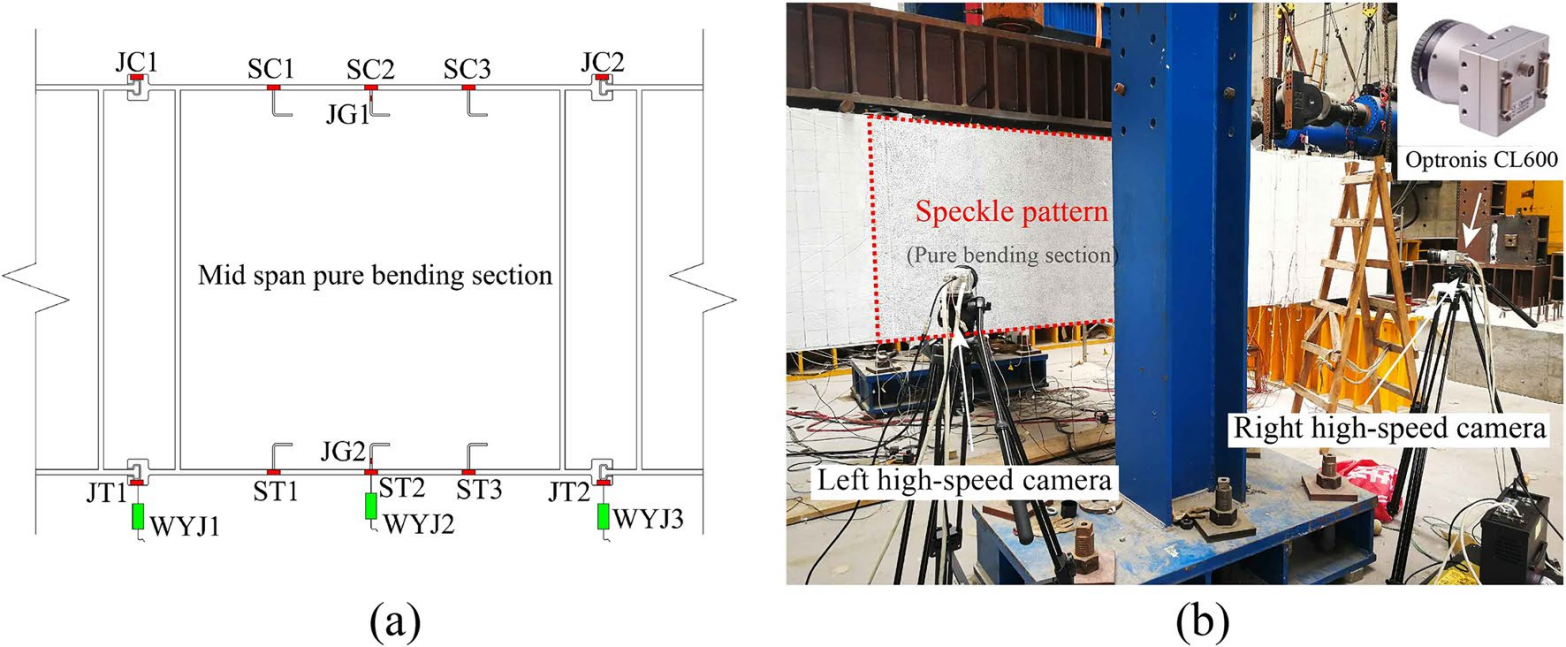
Measurements of the bending test: (**a**) the layout of strain gauges and displacement meters, and (**b**) the binocular vision camera system.

As shown in Fig. 15a, displacement meters WYJ1, WYJ2, and WYJ3 are arranged at the lower edge of the CT-shaped integrated joints under tension at the pure bending section and at the mid-span of the steel plate under tension to monitor the deflection of the specimen. Steel plates, under tension and compression at the pure bending section, are evenly arranged with the strain gauges SC1, SC2, SC3, ST1, ST2, and ST3 at a spacing of 300 mm to monitor their strain. The strain gauges JG1 and JG2 are arranged on the mid-span surface of the shear connector to consider its possible shear failure. The strain gauges JC1, JC2, JT1, and JT2 are arranged on the surface of the CT-shaped integrated joints at the pure bending section to monitor their damage. As shown in Fig. 15b, the surface of the pure bending section is sprayed with a speckle pattern, and a binocular vision camera system (two Optronis CL600 high-speed cameras) is adopted. Based on videogrammetric measurement technology, the three-dimensional full field strain of the pure bending section is monitored through stereoscopic matching and target tracking to investigate whether the plane section assumption is tenable^25^.

### Finite element model

Considering the influence of steel plate thickness, filled concrete strength, and shear connector spacing on the structure performance, as shown in Table 3, a total of 15 FE models were set, of which FE model W14 is consistent with the bending test specimen.

**Table 3:** FE models setting.

	Parameters		
FE models	Steel plate thickness (mm)	Filled concrete strength grade	Shear connector spacing (mm)
W1	12	C50	–
W2	14	C50	–
W3	16	C50	–
W4	18	C50	–
W5	20	C50	–
W6	22	C50	–
W7	16	C35	300
W8	16	C40	300
W9	16	C45	300
W10	16	C55	300
W11	16	C60	300
W12	16	C50	600
W13	16	C50	400
W14	16	C50	300
W15	16	C50	240

**Figure 16:**
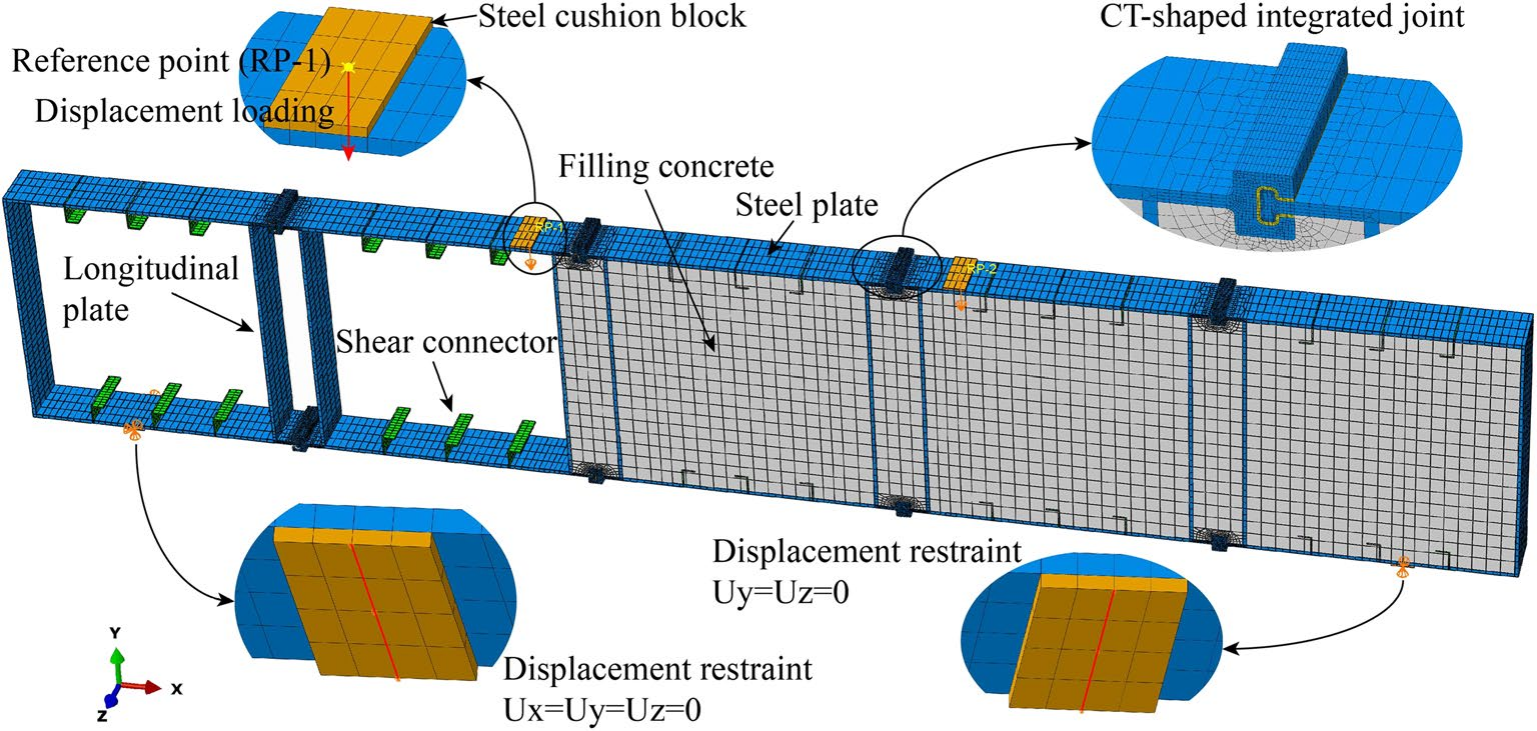
The FE model of W14.

As shown in Fig. 16, establish FE models according to the bending test specimen (Fig. 14). Steel plates, filled concrete, grouting material, and shear connectors are meshed using C3D8R elements (a linear brick element with reduced integration). Steel plates and shear connectors are made of Q235 steel, a nonlinear isotropic/kinematic hardening model in the ABAQUS material library is used for the steel^26^. The mass density of steel material is 7.85 × 10^–9^ g/mm^3^. For the elastic behavior of steel material, the elastic Young's modulus and Poisson's ratio are defined as 200 GPa and 0.3. For the plastic behavior of steel material, the yield strength, ultimate strength and plastic Young's modulus are defined as 235 MPa, 420 MPa and 2 GPa. The constitutive model of filled concrete and grouting material adopts the concrete damage plasticity model in the ABAQUS material library. The mass density of concrete material is 2.5 × 10^–9^ g/mm^3^. The Young's modulus ranges from 31.5 to 36.0 GPa, and the Poisson’s ratio is 0.2. For the concrete damaged plasticity, the dilation angle of 30°, flow potential eccentricity of 0.1, the ratio of biaxial/uniaxial compressive strength of 1.16, invariant stress ratio of 0.667 and viscosity parameter of 0.005 are set respectively.

The interactions among the steel, concrete, and grouting material are simulated by surface-to-surface contact pairs, followed by the hard contact in the normal direction to the interacting surface and the friction coefficient of 0.6 in the tangential direction to the interacting surface^27^. Shear connectors are embedded in the whole model. To prevent stress concentration at the support and loading positions, steel cushion blocks (10 mm thick) are set at the corresponding positions, and the elastic modulus is 100 times that of the steel. The contact between the steel plates and the cushion blocks are constrained by the “Tie” constraint in ABAQUS. FE models adopt the simply supported mode to carry out the four-point bending loading test. The bottom of the steel cushion block located on the left side of the bottom of the structure is restrained against moving in any directions, while the one on the right side is restrained only in Y and Z directions. Two reference points are established on the surfaces of the steel cushion blocks at the top of the structure, and these points come in contact with the blocks by the coupling constraint. By applying vertical displacements to these reference points, the displacement-controlled loading mode is applied, which is conducive to the convergence of FE models.

### Test and FE model results

#### Failure models

**Figure 17:**
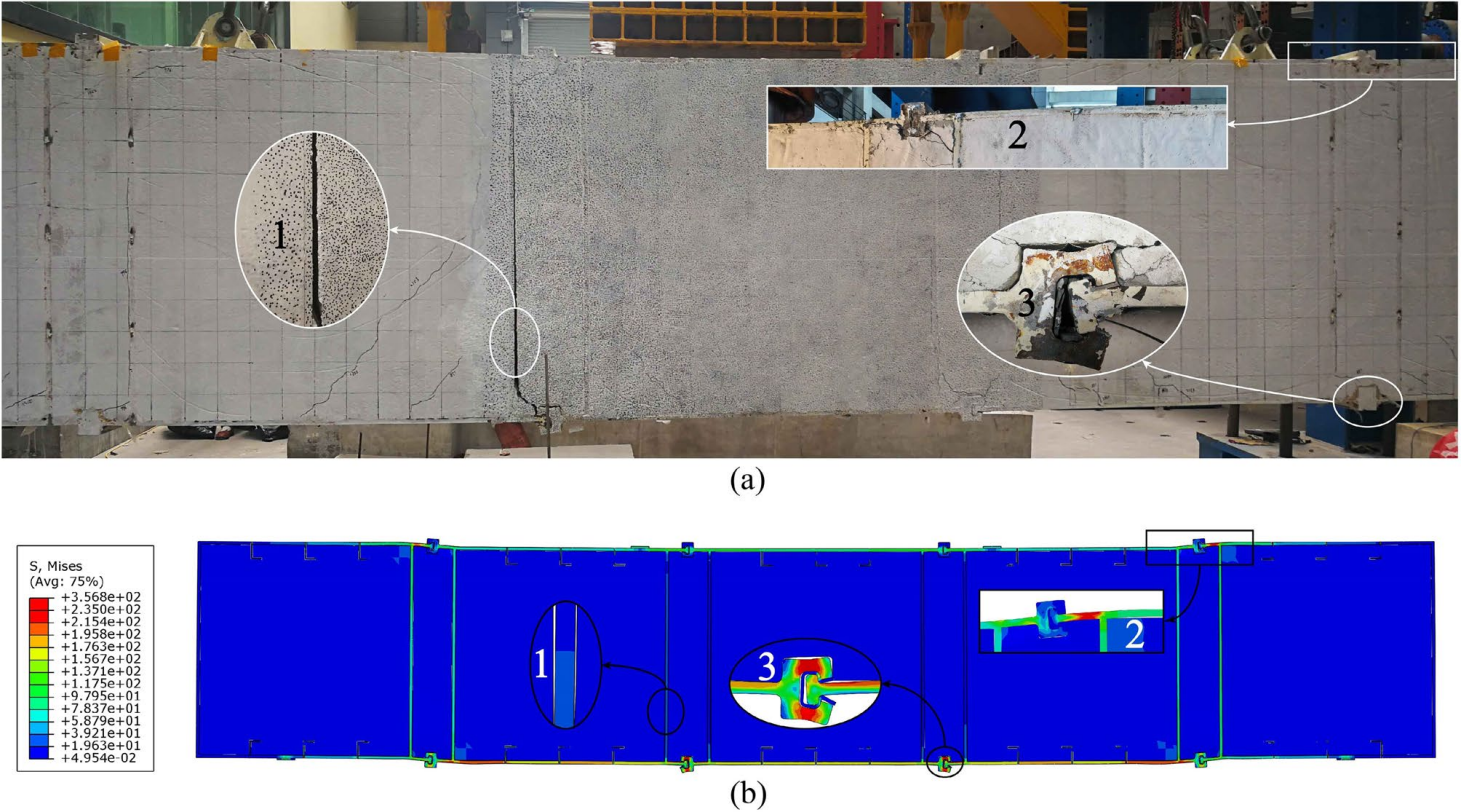
Failure modes of MPJ & JAS tunnel structure: (**a**) bending test results, and (**b**) FE model results of W14.

**Figure 18:**
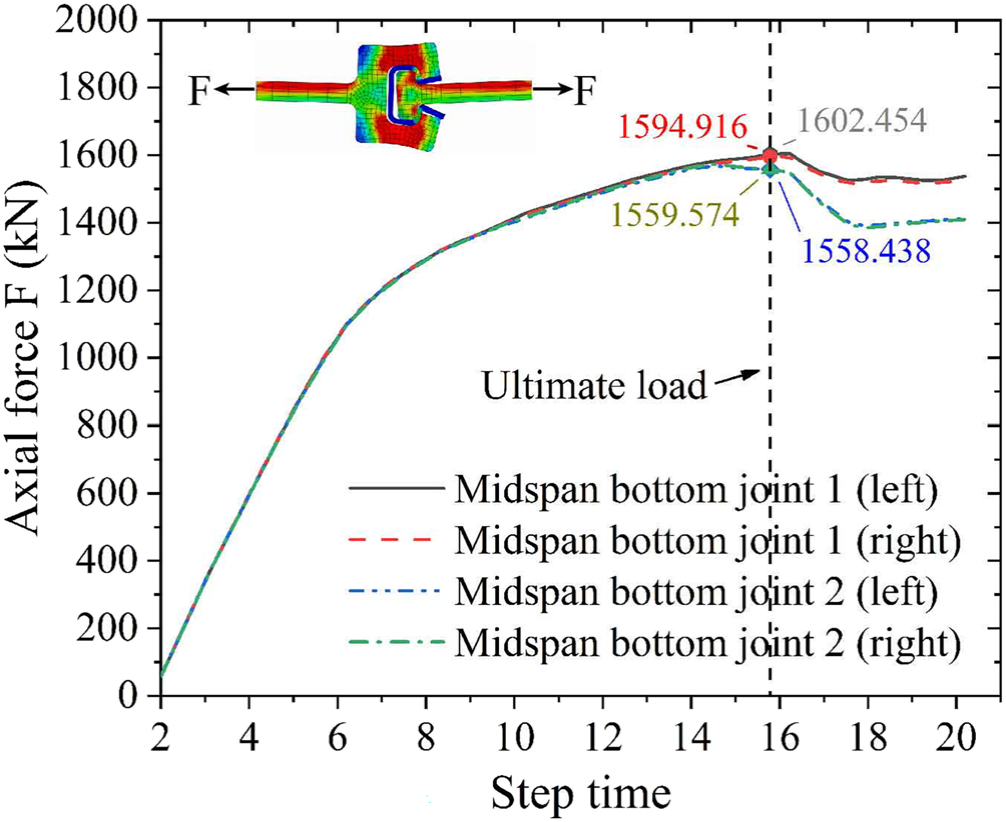
Midspan bottom CT-shaped integrated joints axial forces obtained by FE model results.

Figure 17 shows that the failure models and deformed shapes of the MPJ & JAS tunnel structure of the FE simulation are completely consistent with that of the bending test. The MPJ & JAS tunnel structure shows three typical deformed shapes: shape 1 is the void deformation between the longitudinal plate and the filled concrete, it generates firstly and becomes more serious with the increase of load; shape 2 is the torsional deformation of the top CT-shaped integrated joint under compression, and the compression steel plate tends to buckle slightly with the joint torsion intensifies; shape 3 is the severe tensile failure of the bottom CT-shaped integrated joint under tension, the severe joint deflection deformation results in the grouting material being crushed and the joint finally fail in a state of void and opening. Figure 18 shows that the axial forces of midspan bottom CT-shaped integrated joints when structure fails are 1558.438 kN to 1602.454 kN, which is 194.805–200.307 MPa when converted into the average section stress (section area: 500 mm × 16 mm). The average section stress is almost the same as that of 207.708 MPa in the joint tensile test (tensile strength: 99.7 kN; section area: 30 mm × 16 mm), further demonstrating that the CT-shaped integrated joint is in a tensile failure state. Judging from bending test and FE analysis results, the CT-shaped integrated joint is obviously the weakest part of the MPJ & JAS tunnel structure, and its tensile failure directly induces the structure’s failure.

#### Crack behaviors

**Figure 19:**
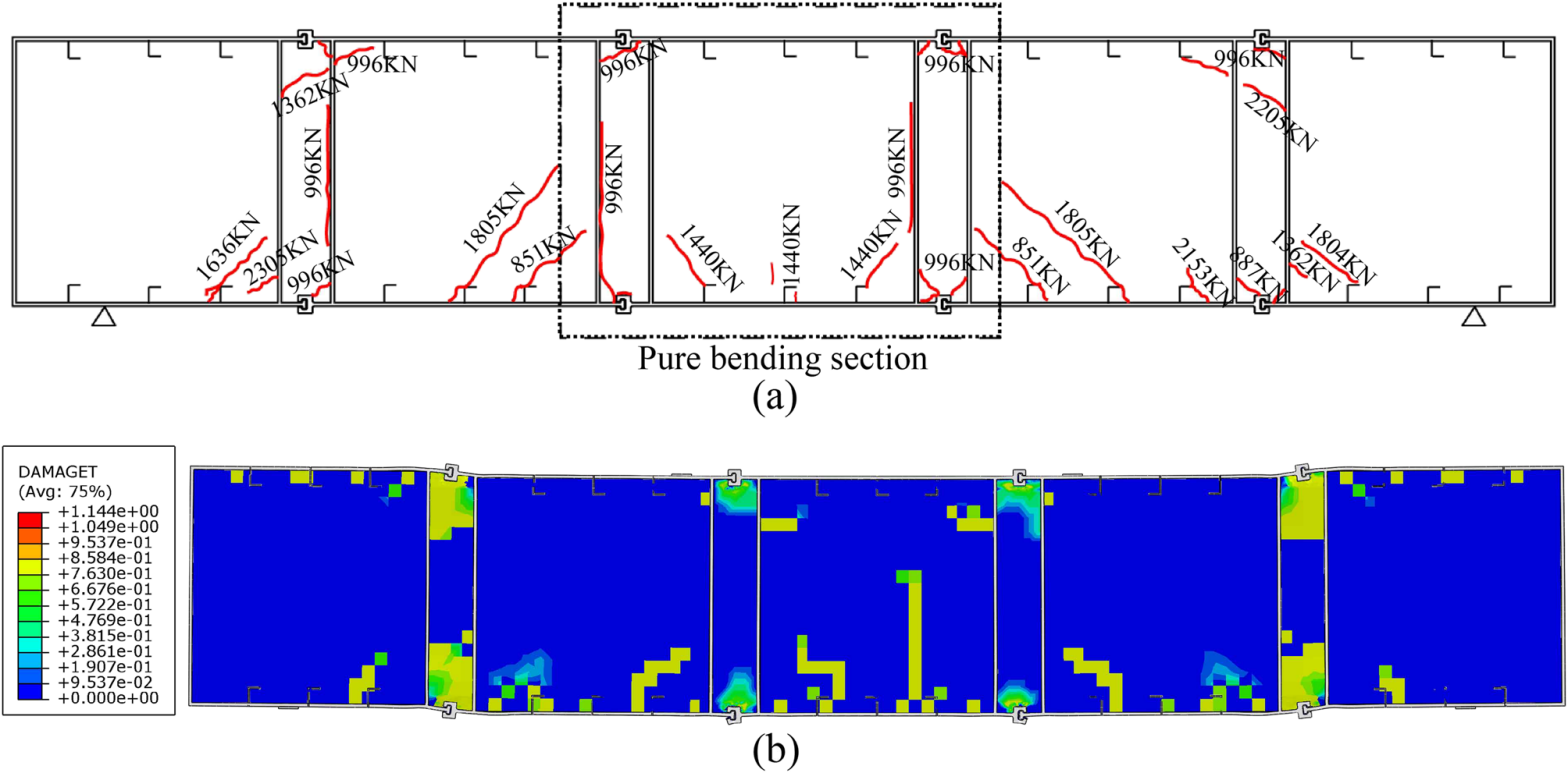
Crack distribution: (**a**) the bending test specimen, and (**b**) the FE model of W14.

According to the bending test, the first crack occurred at the bottom shear connectors under the loading points when loaded to 851 kN. When loaded to 996 kN, cracks were generated from CT-shaped integrated joints and spread to the surrounding. Meanwhile, voids formed between the longitudinal plate and the filled concrete. When loaded to 1440 kN, cracks were generated from the bottom shear connectors of the pure bending section and spread obliquely upward to the loading points. As the loading continues, more and more cracks developed from the outer bottom shear connectors. The experimental crack distribution is shown in Fig. 19a, it was found that almost all cracks were generated from CT-shaped integrated joints and bottom shear connectors due to stress concentration, and those at bottom shear connectors developed obliquely upward in a direction about 45° deviated from the horizontal axis. Figure 19b shows the cracks observed from the FE analysis. The contour plot of the tensile damage variable, i.e., DAMAGET is used to represent the tensile cracks in the concrete. Comparing the crack distribution of test and FE analysis, it shows that the FE model can capture the most of cracks in the concrete.

#### Load–deflection response and stress–strain distribution

**Figure 20:**
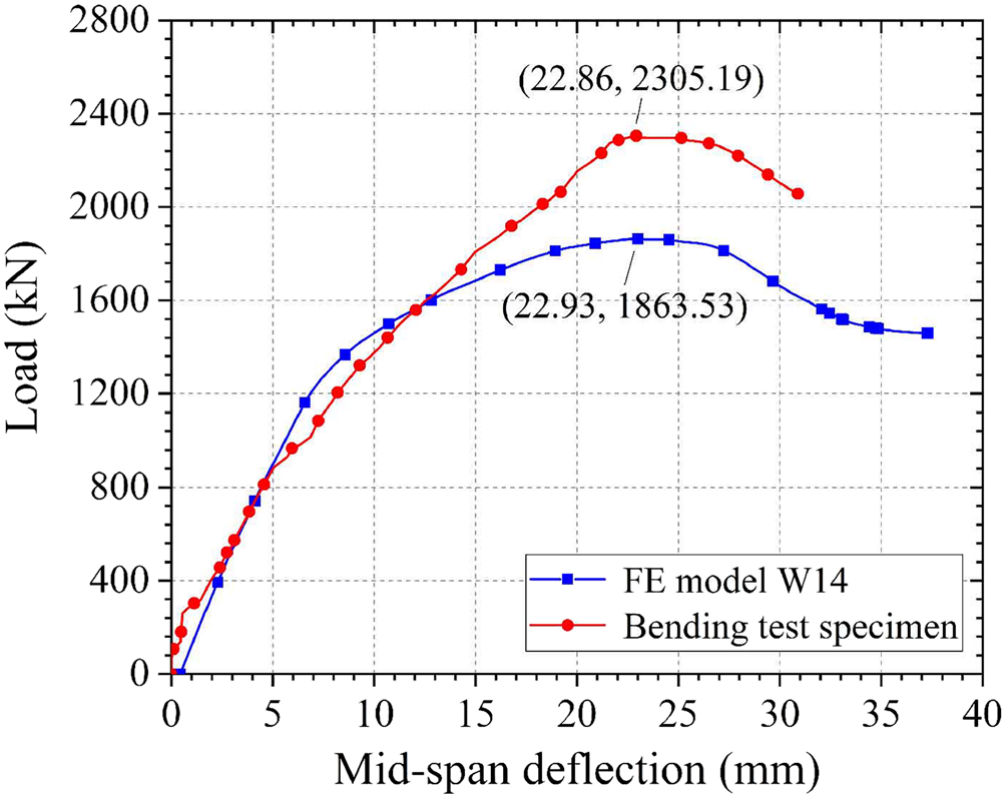
Load–deflection curves of the bending test specimen and the FE model W14.

The load–deflection curves obtained from the bending test specimen and the FE model W14 are generally similar in form, especially in the elastic stage, as is shown in Fig. 20. In the bending test, the mid-span deflection where the structure fails is 22.86 mm; this is only 0.3% different from the 22.93 mm result from the FE analysis, indicating that the FE analysis can effectively predict the structural deformation. However, the ultimate load obtained from the bending test and the FE analysis are 2305.19 and 1863.53 kN, respectively (a difference of 19%). This is mainly because the FE analysis does not consider the characteristics of the high-performance grouting material. Therefore, the strength of the grouting material is underestimated, resulting in a decrease in the strength of the CT-shaped integrated joint and causing a conservative ultimate load in the FE analysis. Overall, the bending test result verifies the reliability of the FE analysis.

**Figure 21:**
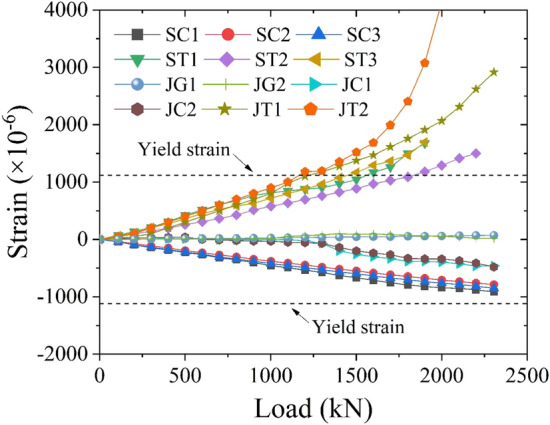
Strain distribution of the bending test specimen.

**Figure 22:**
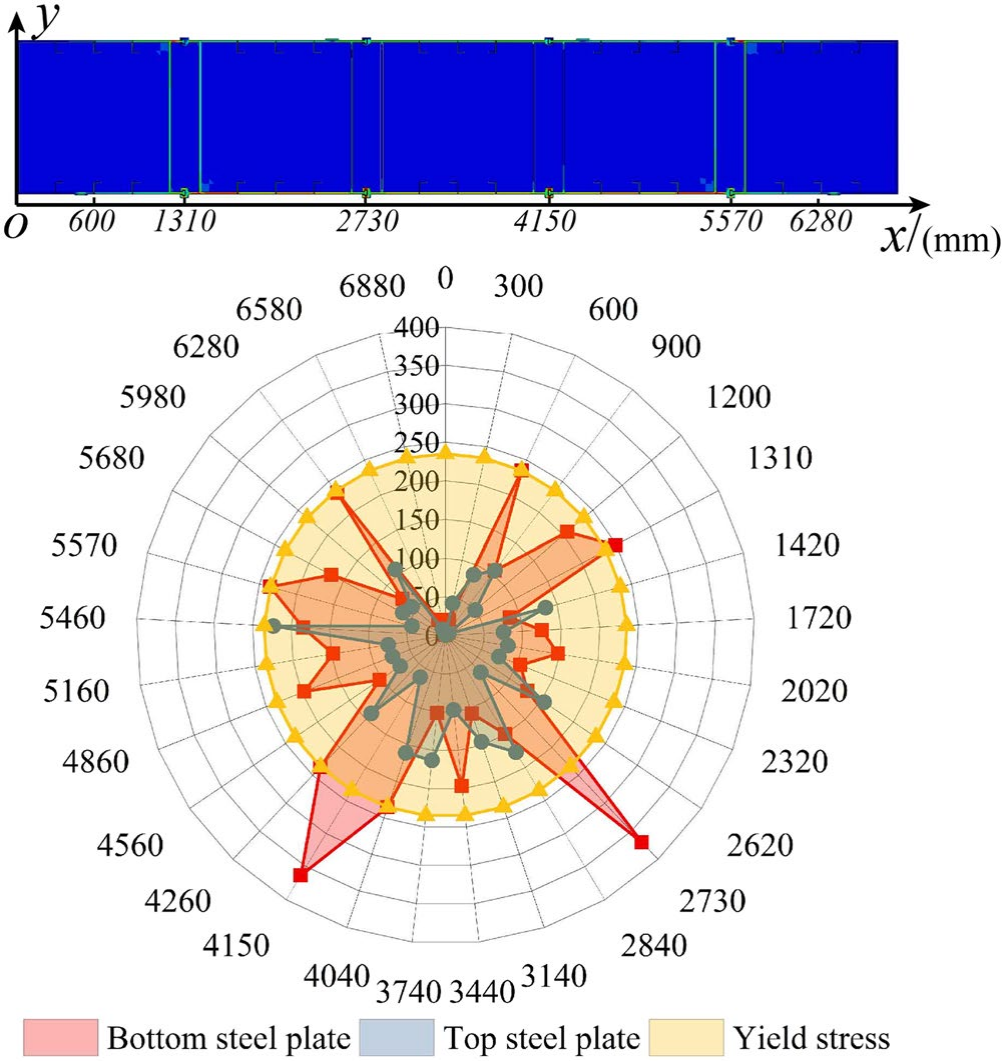
Stress distribution of the FE model W14.

The strain measurement results of the bending test are shown in Fig. 21. The top steel plate (under compression) does not yield when the structure fails, and the bottom steel plate (under tension) begin to yield, but the yield strain remains small. The top CT-shaped integrated joints of the pure bending section do not yield, and the strain is small. However, the bottom CT-shaped integrated joints of the pure bending section do yield, and the yield strain is large. The shear connections do not yield, and the strains are almost negligible. As shown in Fig. 22, the stress distribution of the FE results can more clearly show that the stress of the top steel plate on both sides of the joint is generally greater than that of the top CT-shaped integrated joint, and neither of them yields. Conversely, the stress of the bottom CT-shaped integrated joint is generally greater than that of the bottom steel plate, all bottom CT-shaped integrated joints and midspan bottom steel plate yield, especially the yield stress of the midspan joints is serious. The strain distribution results of bending test and the stress distribution results of FE model are mutually corroborated. This stress–strain distribution is consistent with the failure modes of torsional deformation of compression joints and tensile failure of tension joints, indicating that special attention should be paid to the improvement of CT-shaped integrated joint performance in order to improve the MPJ & JAS tunnel structure performance.

**Figure 23:**
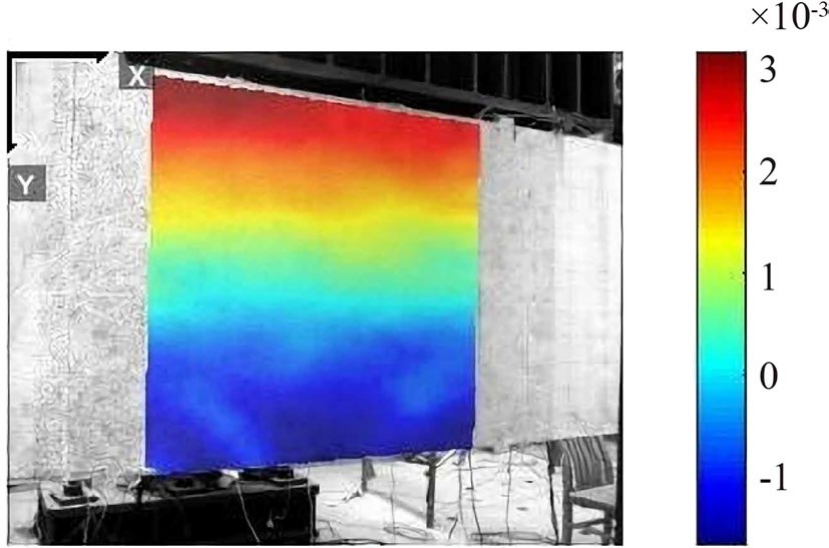
The strain measurement result of the pure bending section.

According to the videogrammetric measurement result shown in Fig. 23, it found that the strain field distribution in the pure bending section is basically linear along the section height, indicating that the plane section assumption is tenable.

#### Influence parameters

The failure models, crack behaviors, load–deflection response, and stress–strain distribution of the FE model W14 agreed with the bending test results. Hence, FE models can be used to analyze the influences of steel plate thickness, filled concrete strength, and shear connector spacing on the MPJ & JAS tunnel structure performance.

**Figure 24:**
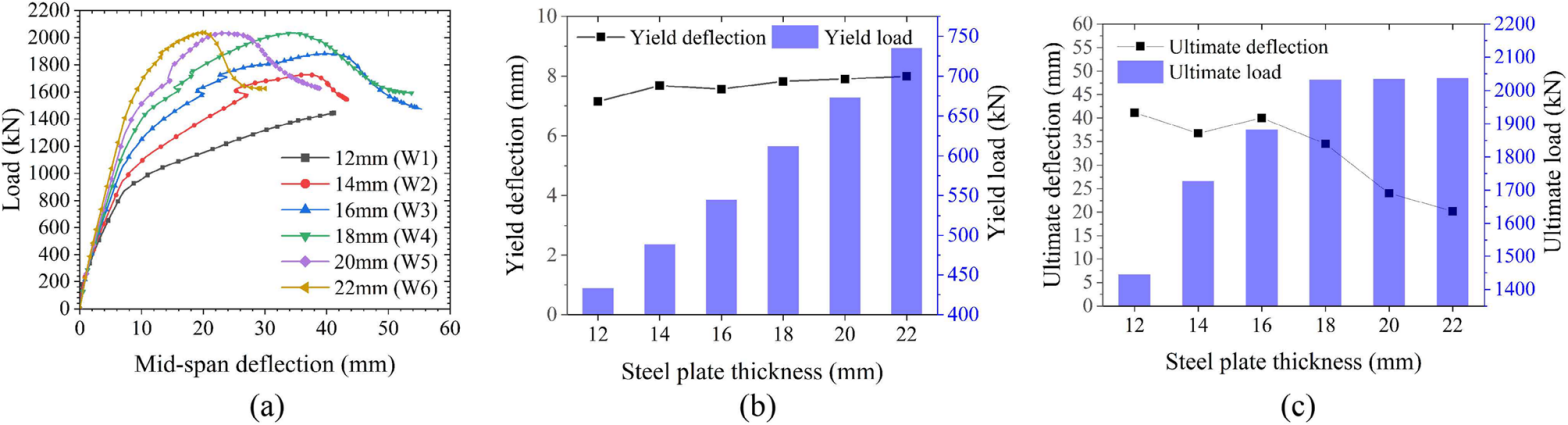
Influences of steel plate thickness on the bearing performance of the MPJ & JAS tunnel structure: (**a**) the load–deflection curves, (**b**) the yield state, and (**c**) the ultimate state.

The influences of the steel plate thickness are shown in Fig. 24. The yield load increases linearly as the steel plate thickness increases, but the steel plate thickness exhibits little effect on the yield deflection. When the steel plate thickness is small (≤ 16 mm), the structure fails due to excessive buckling deformation of the steel plate. Increasing the steel plate thickness improves the ultimate load to a certain extent, as the ultimate load increases linearly along with the increase in steel plate thickness. However, when the steel plate thickness is large (> 16 mm), the ultimate load is mainly controlled by the performance of the CT-shaped integrated joint because the failure of the joint precedes the buckling failure of the steel plate. Therefore, as the steel plate thickness continues to increase, the ultimate load increases only minimally. Little difference is found in the ultimate deflection when the steel plate thickness is lower than 16 mm. However, when the steel plate thickness does exceed 16 mm, the ultimate deflection suddenly decreases linearly, and the structure is in brittle failure. This is primarily due to the increase in structural stiffness as the steel plate thickness increases. In conclusion, it is not necessarily better to have thicker steel plates, a 16 mm thick steel plate is suitable for the MPJ & JAS tunnel structure, ensuring that the requirements of structural bearing capacity and ductile failure are met.

**Figure 25:**
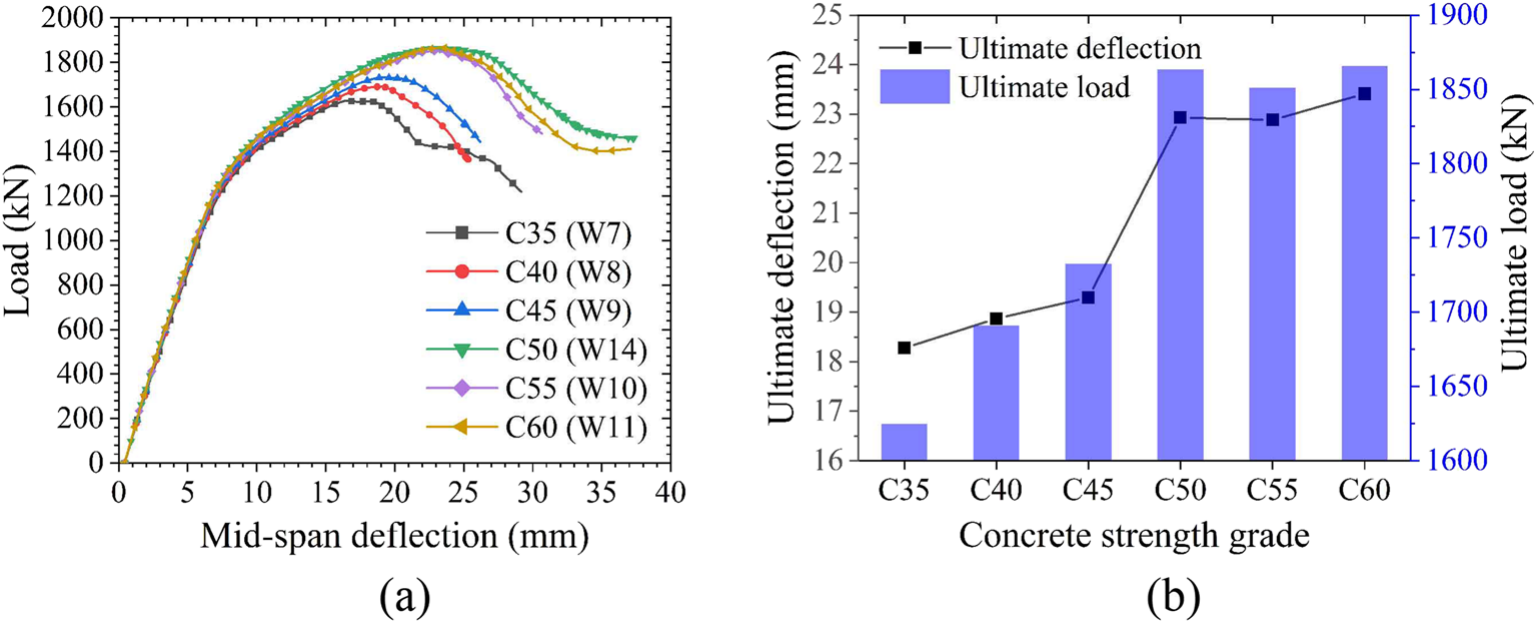
Influences of filled concrete strength on the bearing performance of the MPJ & JAS tunnel structure: (**a**) the load–deflection curves and (**b**) the ultimate state.

The influences of filled concrete strength are shown in Fig. 25. When the filled concrete strength is low (< C50), the tensile steel plate does not yield, and the structure mainly fails by concrete shear failure. Therefore, the ultimate load increases as the filled concrete strength increases. However, when the filled concrete strength is high (≥ C50), the structure fails primarily due to the bending yield of the steel plate, and increasing the filled concrete strength results in little effect on the ultimate load.

**Figure 26:**
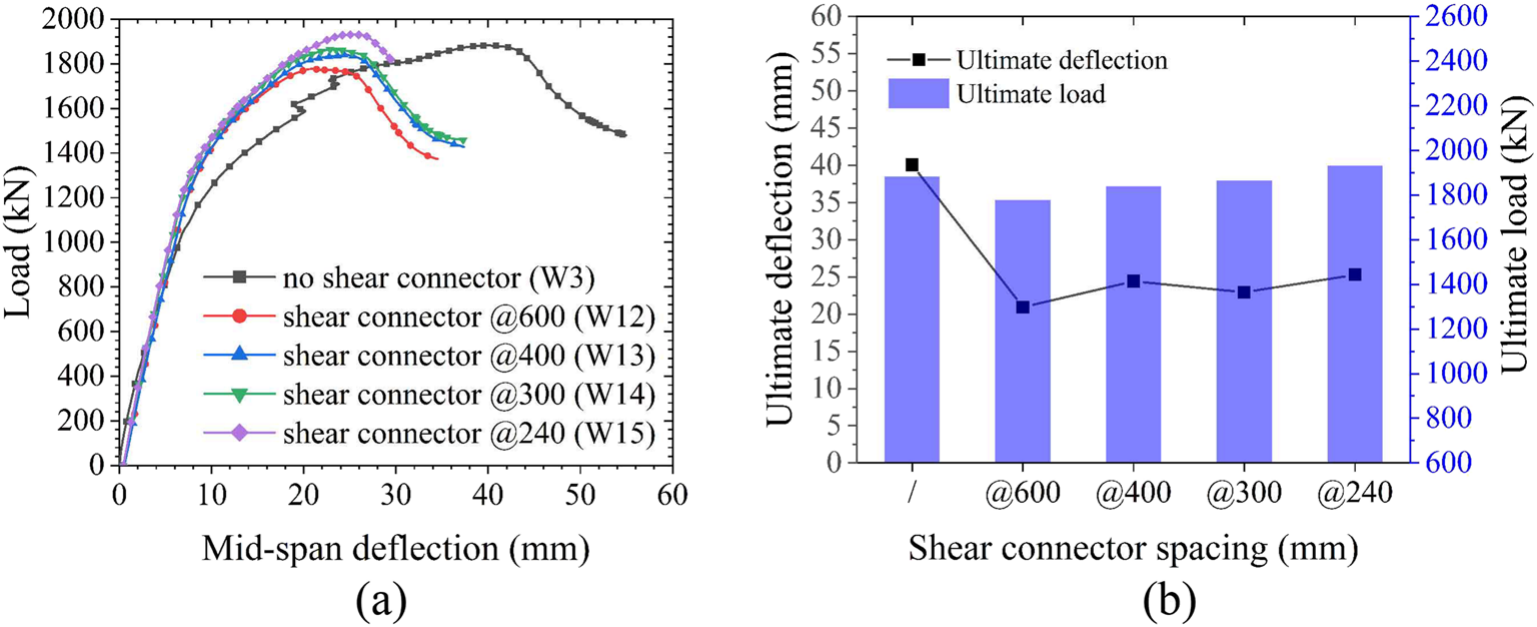
Influences of shear connector spacing on the bearing performance of the MPJ & JAS tunnel structure: (**a**) the load–deflection curves and (**b**) the ultimate state.

The influences of shear connector spacing are shown in Fig. 26. The deflection without the shear connector is large, and the buckling deformation of the steel plate and the void between the steel plate and the filled concrete are severe. The shear connectors can transmit the interface shear force between the steel plate and the concrete as well as provide a strong pull-out resistance; in turn, this improves the stability of the steel plate by ensuring that the steel plate and the concrete are closely connected so they work cooperatively. Shear connectors reduce both the degree of buckling deformation of the steel plate and the structural deflection deformation; however, they hardly affect the ultimate load, and the shear connector spacing demonstrates negligible influence on the ultimate load. Therefore, shear connectors can be used as a constructional measure to reduce structural deformation.

## Theoretical design method for MPJ & JAS tunnel structure

In view of the lack of research on the design theory for the MPJ & JAS tunnel structure, a theoretical design method considering the influence of the CT-shaped integrated joint to predict the bending strength of the MPJ & JAS tunnel structure was proposed in this section based on the CT-shaped integrated joint tensile test results in “Joint tensile test” section and the bending test results in “Test and FE model results” section.

### Assumptions of the theoretical design method

According to the results of the bending test, the following assumptions are adopted in the derivation of the theoretical design method for the prediction of the bending strength of the MPJ & JAS tunnel structure. (1) The section deformation conforms to the plane section assumption. (2) Due to the cohesive force and the pull-out resistance of the shear connectors, no void and slip is found between the steel plate at the upper and lower edges and the filled concrete. Therefore, it is considered that the steel plate and the filled concrete are stressed and deformed together, and the interfacial strain is continuous. (3) The results of the bending test show that the shear connector is intact, and its strain is small; therefore, the contribution of the shear connector to the bending strength is ignored. (4) Under compression, the steel plate does not yield. (5) When the structure fails, only the concrete around CT-shaped integrated joints in the compression zone cracks; therefore, it is considered that the concrete in the compression zone is in an elastic–plastic state, and the concrete stress at the edge of the compression zone does not exceed the axial compressive strength of the concrete. (6) The concrete cracking in the tensile zone is not serious and exhibits a certain bearing capacity; therefore, the model of the tensile zone of the concrete can be simplified into the form of triangle–rectangle–triangle according to the tensile constitutive relationship. (7) To consider the influence of the CT-shaped integrated joint, the weakest part of the MPJ & JAS tunnel structure, the critical condition of the structure failure is defined as the CT-shaped integrated joint reaches the ultimate tensile stress according to the failure phenomenon that the structure reaches the ultimate bearing capacity due to the failure of CT-shaped integrated joints in the bending test. Therefore, the strain of the steel plate under tension, when the structure fails, is equivalent to the ultimate tensile strain of the CT-shaped integrated joint.

### Derivation of the theoretical design method

**Figure 27:**
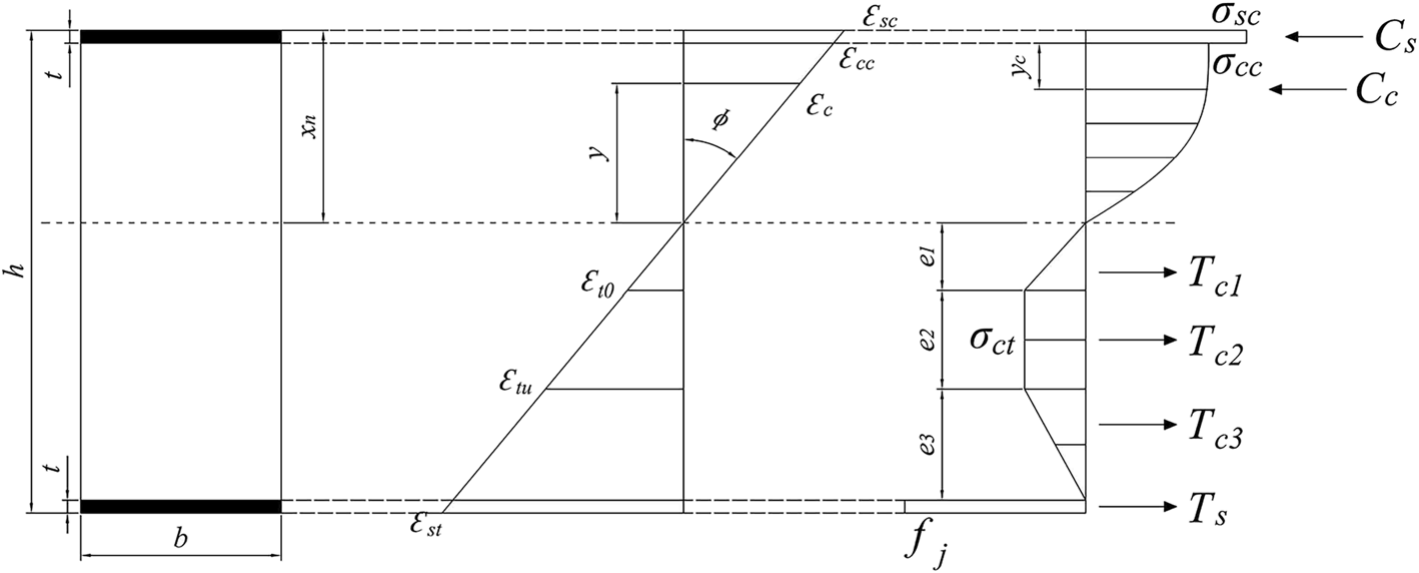
The schematic diagram of the theoretical analysis.

Based on the above assumptions, the schematic diagram of the theoretical analysis is shown in Fig. 27. In the following equations, *h* is the height of the section, *b* is the width of the section, *t* is the thickness of the steel plate, *x*_*n*_ is the height of the neutral axis of the section, and *ϕ* is the curvature of the section.

The ideal elastic–plastic constitutive relationship is selected for the steel, and the constitutive relationship of the concrete is based on the curve recommended in the Chinese code for the design of concrete structures^28^. The stress–strain relationship of the concrete under compression is expressed as follows:

1 $$\left\{ {\begin{array}{*{20}l} {\sigma_{c} = f_{c} \left[ {1 - \left( {1 - {{\varepsilon_{c} } \mathord{\left/ {\vphantom {{\varepsilon_{c} } {\varepsilon_{0} }}} \right. \kern-0pt} {\varepsilon_{0} }}} \right)^{n} } \right]} \hfill & {\varepsilon_{c} \le \varepsilon_{0} } \hfill \\ {\sigma_{c} = f_{c} } \hfill & {\varepsilon_{0} \le \varepsilon_{c} \le \varepsilon_{cu} } \hfill \\ \end{array} } \right.$$

2 $$n = 2 - {{\left( {f_{cu} - 50} \right)} \mathord{\left/ {\vphantom {{\left( {f_{cu} - 50} \right)} {60}}} \right. \kern-0pt} {60}}$$

3 $$\varepsilon_{0} = 0.002 + 0.5\left( {f_{cu} - 50} \right) \times 10^{ - 5}$$

4 $$\varepsilon_{cu} = 0.0033 - \left( {f_{cu} - 50} \right) \times 10^{ - 5}$$

Here, *σ*_*c*_ is the compressive stress of the concrete, *ε*_*c*_ is the compressive strain of the concrete, *f*_*c*_ is the axial compressive strength of the concrete, *ε*_*0*_ is the compressive strain of the concrete when the compressive stress is equal to *f*_*c*_, *ε*_*cu*_ is the ultimate compressive strain of the concrete (when the calculated *ε*_*cu*_ is greater than 0.0033, it is taken as 0.0033), *f*_*cu*_ is the cube strength of the concrete, and *n* is the coefficient (when the calculated *n* is greater than 2.0, it is taken as 2.0).

The stress–strain relationship of the concrete under tension is expressed as follows^29^:

5 $$\left\{ {\begin{array}{*{20}l} {\sigma_{t} = f_{t} \varepsilon_{t} /\varepsilon_{t0} } \hfill & {\varepsilon_{t} < \varepsilon_{t0} } \hfill \\ {\sigma_{t} = f_{t} } \hfill & {\varepsilon_{t} \ge \varepsilon_{t0} } \hfill \\ \end{array} } \right.$$

where *σ*_*t*_ is the tensile stress of the concrete, *ε*_*t*_ is the tensile strain of the concrete, *f*_*t*_ is the axial tensile strength of the concrete, *ε*_*t0*_ is the tensile strain of the concrete when the tensile stress is equal to *f*_*t*_, and *ε*_*tu*_ is the ultimate tensile strain of the concrete. Generally, *ε*_*tu*_ = 2*ε*_*t0*_ and *ε*_*tu*_ = 0.0001.

The parameters of CT-shaped integrated joints are defined as follows. The term *P*_*j*_ is the ultimate tensile strength obtained from the tensile test in “Joint tensile test” section, *b*_*j*_ is the width of the integrated joint, *t*_*j*_ is the thickness of the integrated joint, and *E*_*j*_ is the elastic modulus of the CT-shaped integrated joint material. The ultimate tensile stress *f*_*j*_ and the ultimate tensile strain *ε*_*st*_ of the CT-shaped integrated joint are calculated as follows:

6 $$f_{j} = {{P_{j} } \mathord{\left/ {\vphantom {{P_{j} } {\left( {b_{j} t_{j} } \right)}}} \right. \kern-0pt} {\left( {b_{j} t_{j} } \right)}}$$

7 $$\varepsilon_{st} = {{f_{j} } \mathord{\left/ {\vphantom {{f_{j} } {E_{j} }}} \right. \kern-0pt} {E_{j} }}$$

According to the plane section assumption^29^, the strain at the edge of the concrete compression zone *ε*_*cc*_, the strain of the steel plate under compression *ε*_*sc*_, and the height of the simplified mode of the concrete tensile zone *e*_*1*_, *e*_*2*_, and *e*_*3*_ (corresponding to *ε*_*t0*_ and *ε*_*tu*_) are calculated from *ε*_*st*_, as follows:

8 $$\phi = {{\varepsilon_{sc} } \mathord{\left/ {\vphantom {{\varepsilon_{sc} } {x_{n} }}} \right. \kern-0pt} {x_{n} }} = {{\varepsilon_{cc} } \mathord{\left/ {\vphantom {{\varepsilon_{cc} } {\left( {x_{n} - t} \right)}}} \right. \kern-0pt} {\left( {x_{n} - t} \right)}} = {{\varepsilon_{st} } \mathord{\left/ {\vphantom {{\varepsilon_{st} } {\left( {h - x_{n} } \right)}}} \right. \kern-0pt} {\left( {h - x_{n} } \right)}} = {{\varepsilon_{t0} } \mathord{\left/ {\vphantom {{\varepsilon_{t0} } {e_{1} }}} \right. \kern-0pt} {e_{1} }} = {{\varepsilon_{tu} } \mathord{\left/ {\vphantom {{\varepsilon_{tu} } {\left( {e_{1} + e_{2} } \right)}}} \right. \kern-0pt} {\left( {e_{1} + e_{2} } \right)}}$$

9 $$e_{3} = \left( {h - x_{n} } \right) - \left( {e_{1} + e_{2} } \right) - t$$

The resultant force of the steel plate under tension *T*_*s*_ and the resultant force of the concrete in the tensile zone *T*_*c*_ are calculated as follows:

10 $$T_{s} = f_{j} bt$$

11 $$\left\{ {\begin{array}{*{20}l} {T_{c1} = {{f_{t} e_{1} b} \mathord{\left/ {\vphantom {{f_{t} e_{1} b} 2}} \right. \kern-0pt} 2}} \hfill \\ {T_{c2} = f_{t} e_{2} b} \hfill \\ {T_{c3} = {{f_{t} e_{3} b} \mathord{\left/ {\vphantom {{f_{t} e_{3} b} 2}} \right. \kern-0pt} 2}} \hfill \\ \end{array} } \right.$$

12 $$T_{c} = T_{c1} + T_{c2} + T_{c3}$$

When the concrete strength grade does not exceed C50 (*n* = 2) and *ε*_*cc*_ < *ε*_*0*_,

13 $$\sigma_{c} = f_{c} \left( {{{2\varepsilon_{c} } \mathord{\left/ {\vphantom {{2\varepsilon_{c} } {\varepsilon_{0} }}} \right. \kern-0pt} {\varepsilon_{0} }} - {{\varepsilon_{c}^{2} } \mathord{\left/ {\vphantom {{\varepsilon_{c}^{2} } {\varepsilon_{0}^{2} }}} \right. \kern-0pt} {\varepsilon_{0}^{2} }}} \right)$$

The resultant force of the concrete in compression zone *C*_*c*_ and the distance from its action point to the edge of the concrete compression zone *y*_*c*_ are calculated as follows:

14 $$C_{c} = f_{c} b\int_{0}^{{x_{n} - t}} {\left( {{{2\varepsilon_{c} } \mathord{\left/ {\vphantom {{2\varepsilon_{c} } {\varepsilon_{0} }}} \right. \kern-0pt} {\varepsilon_{0} }} - {{\varepsilon_{c}^{2} } \mathord{\left/ {\vphantom {{\varepsilon_{c}^{2} } {\varepsilon_{0}^{2} }}} \right. \kern-0pt} {\varepsilon_{0}^{2} }}} \right)dy} = f_{c} b\left( {x_{n} - t} \right)\left[ {{{\varepsilon_{cc} } \mathord{\left/ {\vphantom {{\varepsilon_{cc} } {\varepsilon_{0} }}} \right. \kern-0pt} {\varepsilon_{0} }} - {{\varepsilon_{cc}^{2} } \mathord{\left/ {\vphantom {{\varepsilon_{cc}^{2} } {\left( {3\varepsilon_{0} } \right)^{2} }}} \right. \kern-0pt} {\left( {3\varepsilon_{0} } \right)^{2} }}} \right]$$

15 $$y_{c} = \left( {x_{n} - t} \right) - \frac{{f_{c} b\int_{0}^{{x_{n} - t}} {\left( {{{2\varepsilon_{c} } \mathord{\left/ {\vphantom {{2\varepsilon_{c} } {\varepsilon_{0} }}} \right. \kern-0pt} {\varepsilon_{0} }} - {{\varepsilon_{c}^{2} } \mathord{\left/ {\vphantom {{\varepsilon_{c}^{2} } {\varepsilon_{0}^{2} }}} \right. \kern-0pt} {\varepsilon_{0}^{2} }}} \right)ydy} }}{{f_{c} b\int_{0}^{{x_{n} - t}} {\left( {{{2\varepsilon_{c} } \mathord{\left/ {\vphantom {{2\varepsilon_{c} } {\varepsilon_{0} }}} \right. \kern-0pt} {\varepsilon_{0} }} - {{\varepsilon_{c}^{2} } \mathord{\left/ {\vphantom {{\varepsilon_{c}^{2} } {\varepsilon_{0}^{2} }}} \right. \kern-0pt} {\varepsilon_{0}^{2} }}} \right)dy} }} = \left( {x_{n} - t} \right)\frac{{{1 \mathord{\left/ {\vphantom {1 3}} \right. \kern-0pt} 3} - {{\varepsilon_{cc} } \mathord{\left/ {\vphantom {{\varepsilon_{cc} } {\left( {12\varepsilon_{0} } \right)}}} \right. \kern-0pt} {\left( {12\varepsilon_{0} } \right)}}}}{{1 - {{\varepsilon_{cc} } \mathord{\left/ {\vphantom {{\varepsilon_{cc} } {\left( {3\varepsilon_{0} } \right)}}} \right. \kern-0pt} {\left( {3\varepsilon_{0} } \right)}}}}$$

The resultant force of the steel plate under compression *C*_*s*_ is calculated as follows:

16 $$C_{s} = E_{s} \varepsilon_{sc} bt = {{f_{j} btx_{n} } \mathord{\left/ {\vphantom {{f_{j} btx_{n} } {\left( {h - x_{n} } \right)}}} \right. \kern-0pt} {\left( {h - x_{n} } \right)}}$$

where *E*_*s*_ is the elastic modulus of the steel plate.

According to the force balance of the section,

17 $$C_{s} + C_{c} = T_{c} + T_{s}$$

After *x*_*n*_ is obtained according to Eq. (17), the moment at the center of the steel plate is taken under compression, and the ultimate bending moment of the section *M*_*u*_ is calculated as follows:

18 $$M_{u} = T_{s} \left( {h - t} \right) + T_{c1} \left( {{{2e_{1} } \mathord{\left/ {\vphantom {{2e_{1} } 3}} \right. \kern-0pt} 3} + x_{n} - {t \mathord{\left/ {\vphantom {t 2}} \right. \kern-0pt} 2}} \right) + T_{c2} \left( {{{e_{1} + e_{2} } \mathord{\left/ {\vphantom {{e_{1} + e_{2} } 2}} \right. \kern-0pt} 2} + x_{n} - {t \mathord{\left/ {\vphantom {t 2}} \right. \kern-0pt} 2}} \right) + T_{c3} \left( {{{e_{1} + e_{2} + e_{3} } \mathord{\left/ {\vphantom {{e_{1} + e_{2} + e_{3} } 3}} \right. \kern-0pt} 3} + x_{n} - {t \mathord{\left/ {\vphantom {t 2}} \right. \kern-0pt} 2}} \right) - C_{c} \left( {y_{c} + {t \mathord{\left/ {\vphantom {t 2}} \right. \kern-0pt} 2}} \right)$$

The ultimate bending strength *P*_*u*_ and the ultimate bending moment of the section *M*_*u*_ are converted as follows:

19 $$P_{u} = {{2M_{u} } \mathord{\left/ {\vphantom {{2M_{u} } a}} \right. \kern-0pt} a}$$

where *a* is the horizontal distance from the loading point to the support.

### Verification of the theoretical design method

In the tensile test of the CT-shaped integrated joint in “Joint tensile test” section, the ultimate tensile strength *P*_*j*_ is 99.70 kN, the width *b*_*j*_ is 30 mm, and the thickness *t*_*j*_ is 16 mm. The material of the CT-shaped integrated joint is Q235 steel, and its elastic modulus *E*_*j*_ is 210 GPa. According to Eqs. (6) and (7), the ultimate tensile stress *f*_*j*_ and the ultimate tensile strain *ε*_*st*_ of the CT-shaped integrated joint are 207.71 MPa and 9.89 × 10^−4^, respectively.

The ultimate bending strength of the specimen in “Test and FE model results” section, calculated according to the theoretical design method proposed in “Derivation of the theoretical design method” section, is 2101.10 kN, and it is 2305.19 kN in the bending test. The ratio of the experimental value to the theoretical value of the ultimate bending strength is 1.10, indicating that the theoretical design method proposed in this paper, considering the influence of the CT-shaped integrated joint on the structure, can reliably predict the bending strength of the MPJ & JAS tunnel structure only based on a simple tensile test of the CT-shaped integrated joint.

In addition, the MPJ & JAS tunnel structure can be regarded as an SCS composite structure with CT-shaped integrated joints. Therefore, the relevant design methods of the SCS composite structure can be used to predict the bending strength of the MPJ & JAS tunnel structure. Here, the technical standard for the steel plate-concrete structure of nuclear power plants in China is taken as an example^30^. This standard stipulates that when the steel plates on both sides of the concrete structure are symmetrically arranged, or the difference in the net sectional area of the plates per unit width of the steel plates on both sides of the structure is no more than 30%, the design value of the bending strength of the steel plate-concrete structure per unit width is calculated as follows:

20 $$M_{u} = 0.9A_{pn1} fZ_{s}$$

where *f* is the design value of the tensile strength of the steel, *A*_*pn1*_ is the net sectional area of the steel plate on the tension side of the steel plate-concrete structure with unit width, and *Z*_*s*_ is the centroid distance between the steel plates on both sides.

A Japanese code also relates to SCS structures^31^. This code simplifies the distance between the centroid of the tensile steel plate and the acting point of the resultant force in the compression area to 0.875 times the section height. Therefore, the bending strength of an SCS structure is calculated as follows:

21 $$M_{u} = {{7A_{s} f_{y} } \mathord{\left/ {\vphantom {{7A_{s} f_{y} } {\left( {8h} \right)}}} \right. \kern-0pt} {\left( {8h} \right)}}$$

where *A*_*s*_ is the sectional area of the tensile steel plate, *f*_*y*_ is the yield strength of the steel plate, and *h* is the height of the section.

The experimental and theoretical values of the ultimate bending strength are shown in Table 4. The error of the ultimate bending strength calculated according to the theoretical design method proposed in this paper is controlled within 10%. However, according to the Chinese standard and the Japanese code, it is large, with an error between 10 and 20%. The main reason for this is that the outer steel plate of the MPJ & JAS tunnel structure is connected by the CT-shaped integrated joint and is not continuous; therefore, the failure behavior of the MPJ & JAS tunnel structure is not completely consistent with an SCS composite structure that exhibits continuous outer steel plates. This causes a certain degree of error in the ultimate bending strength of the MPJ & JAS tunnel structure calculated by the design methods related to the SCS composite structure. Therefore, this paper recommends predicting the bending strength of the MPJ & JAS tunnel structure with the theoretical design method proposed in “Derivation of the theoretical design method” section.

**Table 4:** Comparison of theoretical design methods.

Specimen	Experimental value (kN)	Chinese standard		Japanese code		Proposed theoretical design method	
		Theoretical value (kN)	Error (%)	Theoretical value (kN)	Error (%)	Theoretical value (kN)	Error (%)
Bending test specimen	2305.19	1870.24	− 18.9	2014.29	− 12.6	2101.10	− 8.9

## Preparation works for construction

MPJ & JAS tunnel is an elemental structure, connecting the elements into a whole through special joints. Due to the characteristics of MPJ & JAS tunnel, it is necessary to develop targeted pipe jacking machine and high-precision and high-quality construction technologies to complete the tunnel construction.

### Micro pipe jacking machine

**Figure 28:**
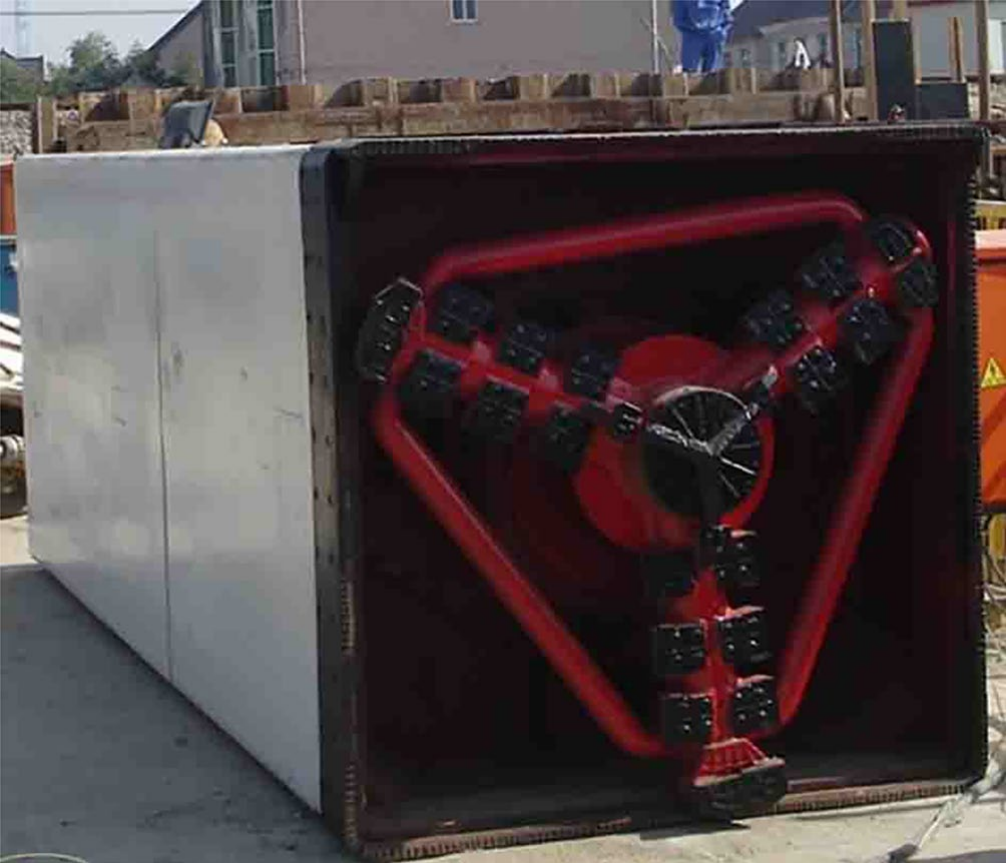
Micro pipe jacking machine.

Micro pipe jacking machine (Fig. 28) is used for the construction of MPJ & JAS method. The main difference between the micro pipe jacking machine and the ordinary shield machine is that the former has a small rectangular section which is very suitable for the jacking of small-sized rectangular steel pipes, with a ratio of about 1:1 and a section size of about 1.2 m × 1.2 m. The main parameters of the micro pipe jacking machine cutterhead are shown in Table 5.

**Table 5:** Parameters of micro pipe jacking machine cutterhead.

Parameters	Cutting power (kW)	Cutterhead speed (r/min)	Driving torque (kN·m)	Torque coefficient (kN/m^2^)	Cutting area ratio (%)
Values	11 × 2	3	70	40.5	100

**Figure 29:**
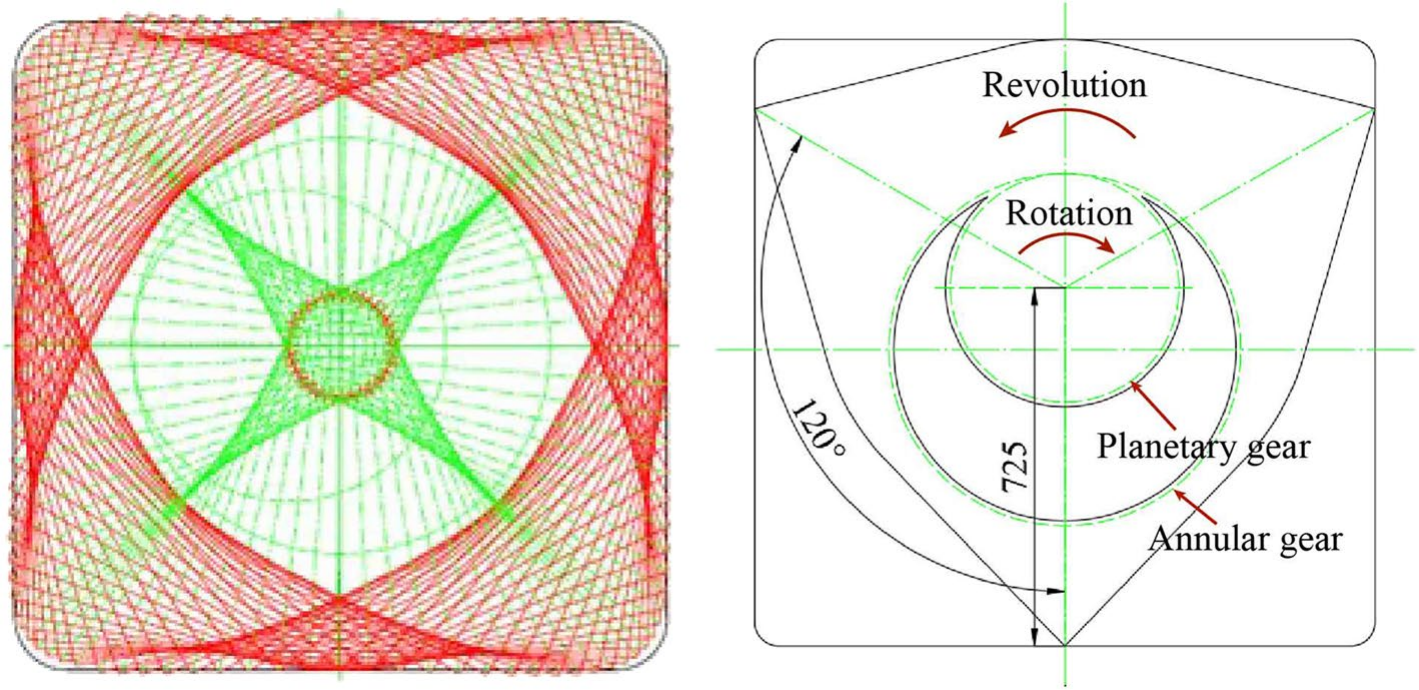
Design of planetary gear for micro pipe jacking machine.

The main characteristics of the micro pipe jacking machine are as follows:

1. Earth pressure balance mode is adopted, screw conveyor is adopted, and frequency conversion is used to automatically control the soil in and out. During jacking, through the fine adjustment of excavation volume, pushing speed and cutter head speed, the automatic earth pressure balance system of the machine head ensures the face stability and reduces the ground settlement.
2. The cutter head is in the form of panel and inner cutter head, greatly reducing the soil disturbance between the steel pipe elements, and achieving accurate jacking construction.
3. The micro pipe jacking machine is of planetary gear type, and the cutting path of the cutter head is close to the rectangle through the cooperation of the revolution and rotation of the internal and external planetary gears (Fig. 29).

### Soil reinforcement

In order to achieve micro disturbance of MPJ & JAS construction method without affecting the normal operation of the existing road, the soil reinforcement measure is necessary. NanoCrete 75 is used to improve the soil mass in the jacking area of steel pipe elements. It is a solvent-free, low viscosity (5 mPa s) hydrophilic Nano Silica Gel Lotion grouting material, composed of amorphous silica. After NanoCrete 75 and the catalyst used with it are mixed evenly in a certain proportion, they are injected into the soil mass through the grouting pump. The setting time (10–150 min) can be controlled by adjusting the proportion of catalyst according to the actual situation of the construction site. The reinforced soil has good impermeability and certain bearing capacity, which can reduce the risk of groundwater, enhance the strength of soil, and reduce the disturbance of jacking construction to the surrounding soil. However, the unconfined compressive strength is low, which is conducive to the cutting of the cutter head of the micro pipe jacking machine, and avoids the cutting difficulty of the pipe jacking machine caused by the excessive strength of the reinforced soil or the excessive posture of the pipe jacking machine caused by the uneven strength of the reinforced soil. After 24–36 h of grouting, the subsequent jacking construction can be carried out.

### Hydraulic traction of reference pipe

**Figure 30:**
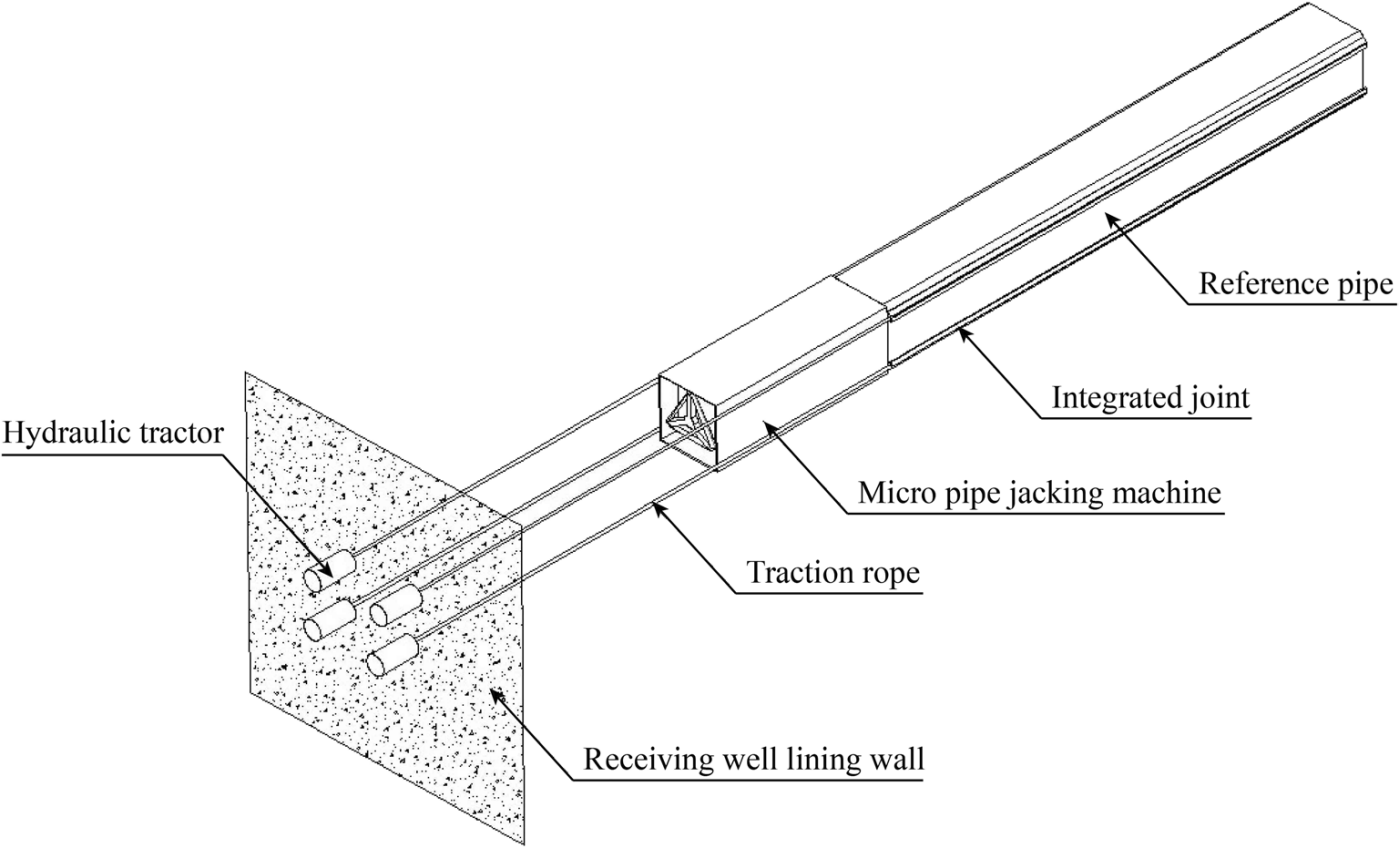
Pilot hydraulic traction system.

**Figure 31:**
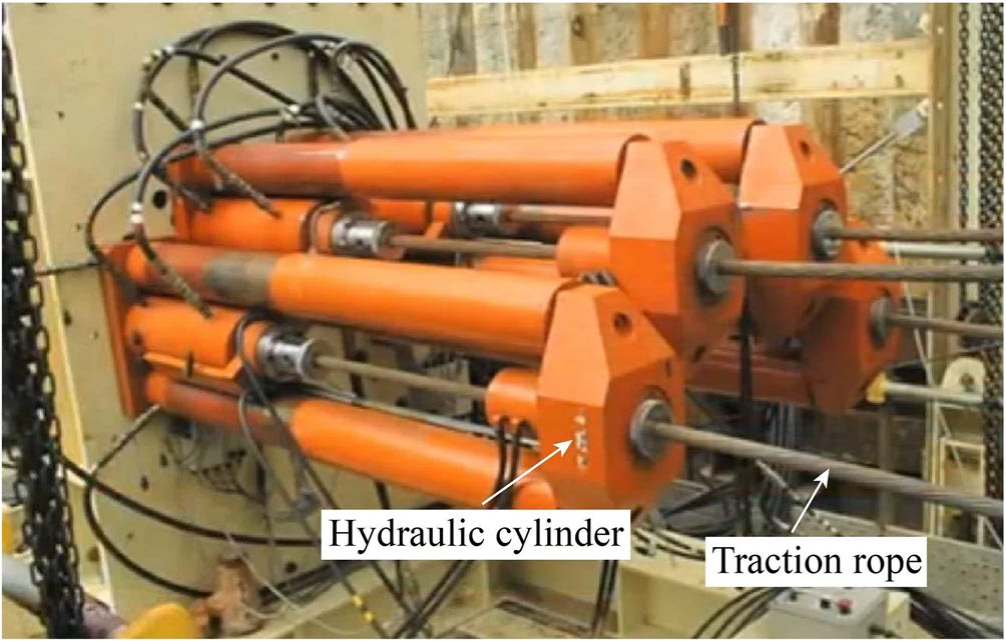
Hydraulic tractor.

**Figure 32:**
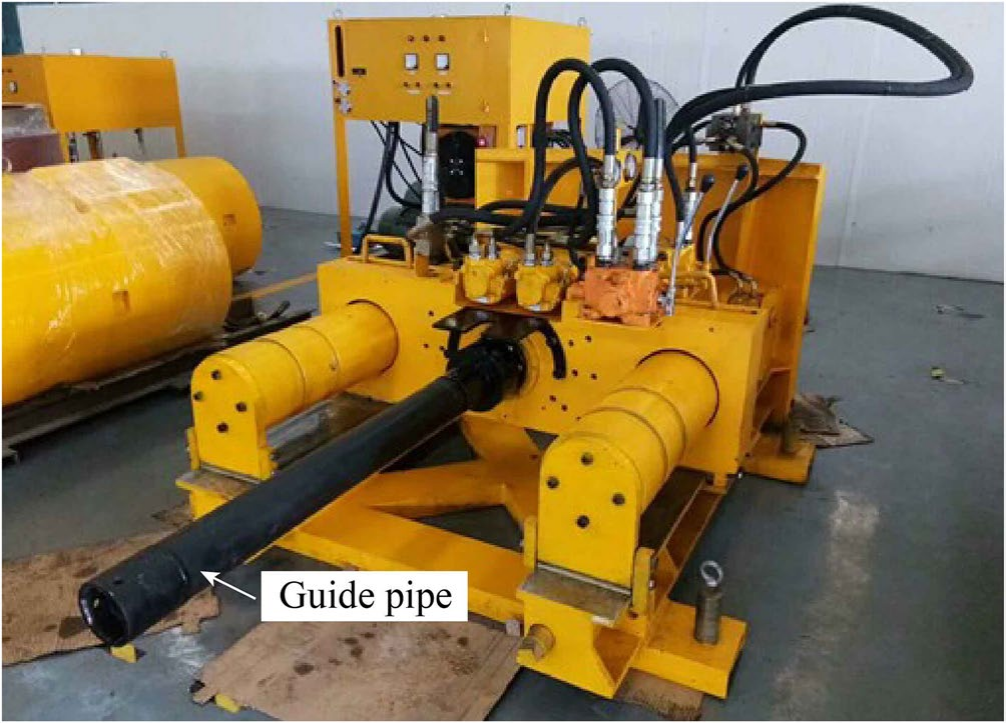
Micro drilling machine.

The traditional pipe jacking method is difficult to solve the jacking attitude problem of the micro pipe jacking machine, and cannot guarantee the construction accuracy of the reference pipe, which will cause difficulties in the subsequent construction of the socket pipe. Pilot hydraulic traction system (Fig. 30) can accurately control the construction of the reference pipe. It uses the hydraulic tractor (Fig. 31) and the traction rope to pull the micro pipe jacking machine. First, four guide pipes with a diameter of 89 mm are jacked by a micro drilling machine (Fig. 32), and the high-strength low relaxation prestressed steel strands with a diameter of 18 mm are passed through them to complete the arrangement of the traction rope. Then, a hydraulic tractor composed of four sets of hydraulic cylinders can be installed on the inner lining wall of the receiving well. The hydraulic cylinder designed with rated load of 2000 kN and rated pressure of 25 MPa can pass through the traction rope. One end of the traction rope is fixed on the hydraulic tractor and the other end is fixed on the micro pipe jacking machine. The hydraulic tractor provides traction power, and through the synchronous control system, the four traction ropes synchronously pull the micro pipe jacking machine to realize the accurate construction of the reference pipe.

### High-precision guidance of pipe roof

**Figure 33:**
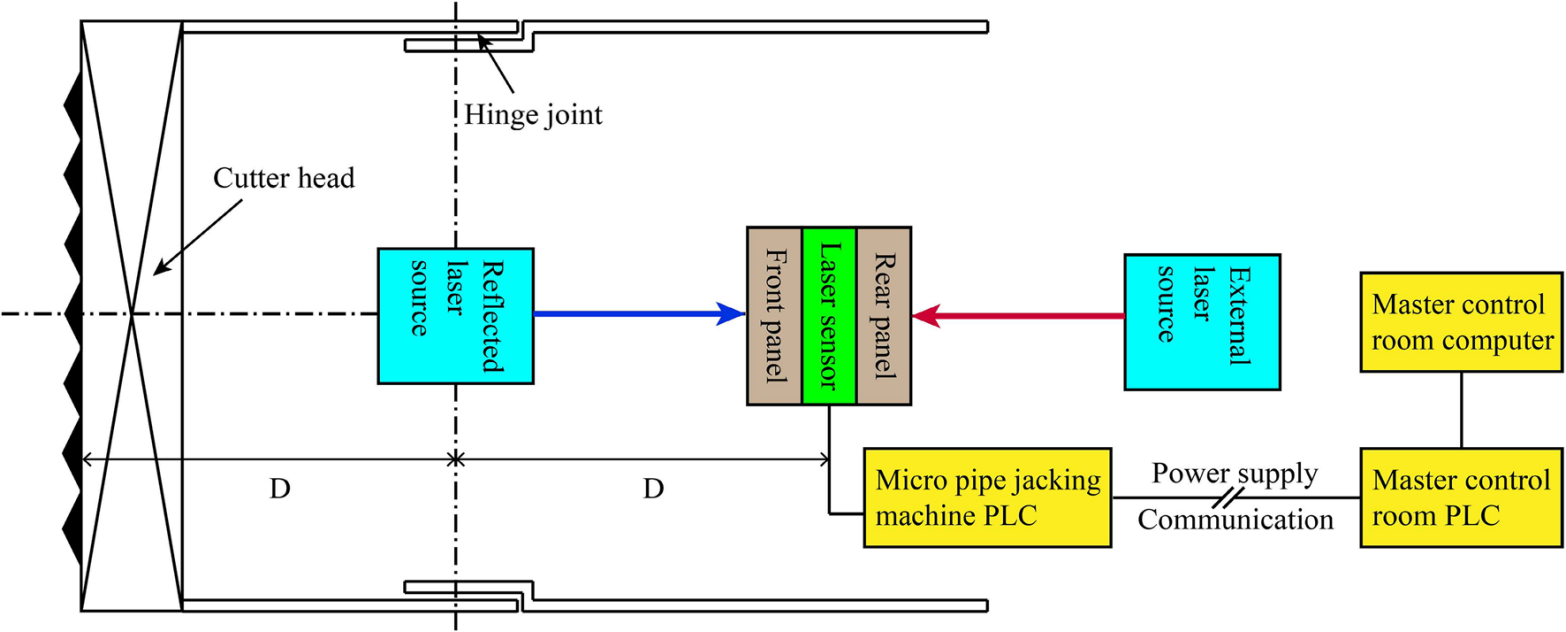
High-precision guidance system.

The attitude deviation control requirements for the whole process of pipe roof jacking are high. Once the deviation exceeds the standard, it is easy to cause the fracture and damage of T-shaped sub joint or the slotting deformation of C-shaped female joint, affecting the socket of steel pipe elements. Therefore, the micro pipe jacking machine needs to be equipped with a high-precision guidance system to assist the pipe roof construction. As shown in Fig. 33, the high-precision guidance system can obtain the axis deviation of the micro pipe jacking machine body when the laser beam of the external laser source in the originating well is irradiated on the rear panel of the laser sensor, providing a quantitative basis for the deviation correction control of steel pipe elements. When the reflected laser beam of the reflected laser source placed at the center of the motor is irradiated on the front panel of the laser sensor, the deviation of the cutter head center can be obtained, providing a quantitative basis for the deviation correction control of the machine head.

**Figure 34:**
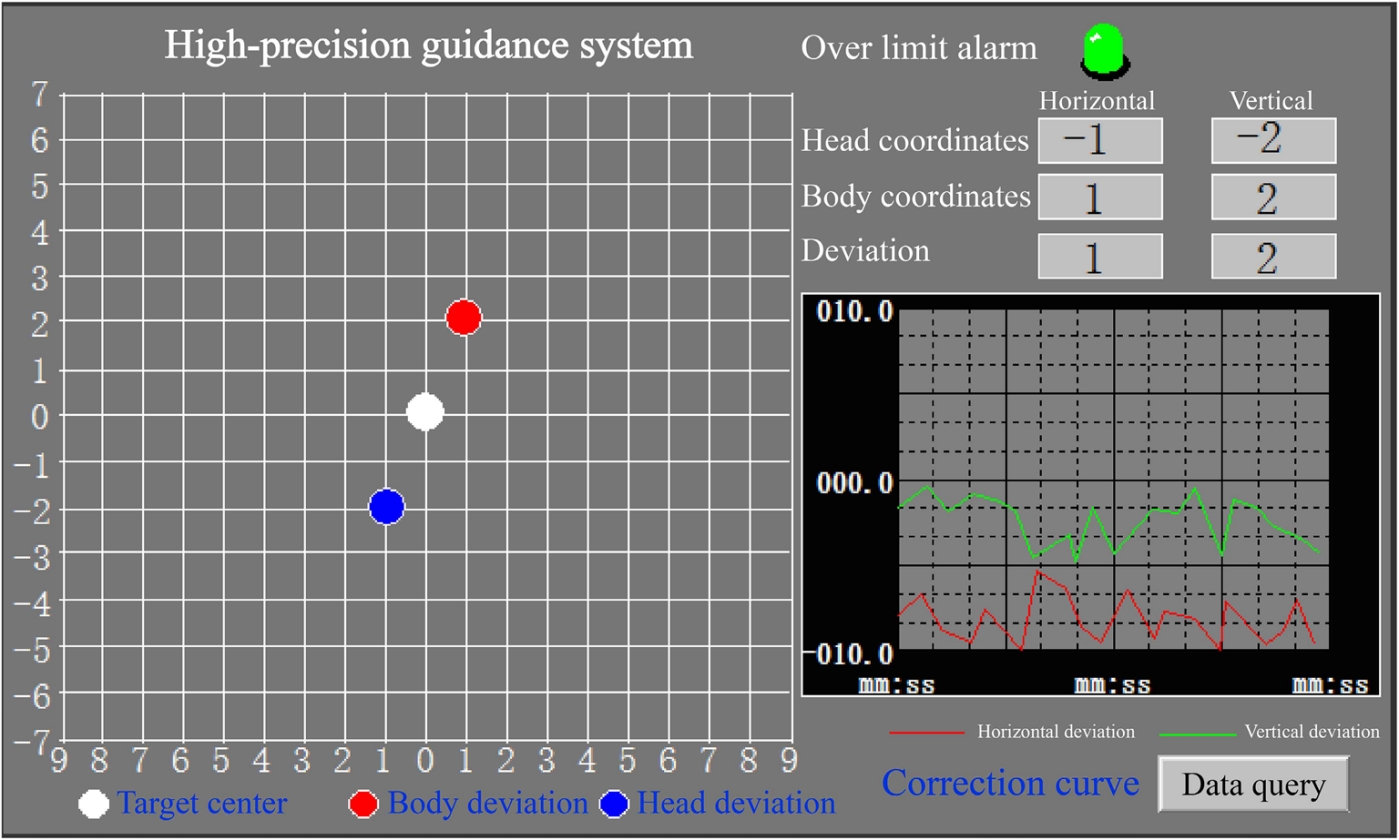
Display interface of the high-precision guidance system.

As shown in Fig. 34, the red dot on the display interface of the high-precision guidance system represents the deviation of the steel pipe element axis, and the blue dot represents the deviation of the pipe jacking machine head center. The operator controls the expansion and contraction of the hinge cylinder through the hinge system to adjust the angle between the front and rear shells of the micro pipe jacking machine and generate the lateral force to correct the deviation. According to the real-time positions of the red, white and blue points, it is constantly adjusted and fitted to accurately correct the deviation, so as to ensure that the attitude deviation of the micro pipe jacking machine is within the control range. In addition, the real-time deviation curve can be generated according to the deviation data stored in the background database. Through the curve trend change, the traveling direction of the micro pipe jacking machine can be more intuitively and effectively mastered, so as to adjust the deviation correction amount in advance and reduce the risk of losing control of posture.

### Concrete construction quality detection

Improper construction of concrete may cause defects such as void between steel pipe and concrete or non-dense pouring of inner concrete, which will seriously affect the structural performance of MPJ & JAS tunnel. The phased array ultrasonic imaging technology^32^ is used to detect concrete defects in MPJ & JAS method. Its basic principle is to change the phase relation when the acoustic wave reaches (or comes from) a certain point in the object by controlling the different delay times of the pulses emitted (or received) by each array element in the transducer array, so as to realize the change of the focus and the direction of the acoustic beam, and then use the synthetic aperture focused ultrasonic imaging technology to display the image signal in real time. When the concrete has defects such as void, hole and non-dense, the interface between the concrete and the air layer will cause acoustic beam reflection and pulse inversion due to the change of medium density. Therefore, concrete defects are displayed as red highlighted reflection areas on the image. Compared with the traditional ultrasonic testing technology, phased array ultrasonic imaging technology can easily be imaged by continuously scanning only one side of the detected object, which is very suitable for MPJ & JAS tunnel with only one free face.

**Figure 35:**
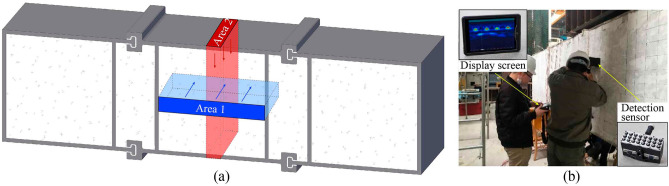
Concrete construction quality detection test based on phased array ultrasonic imaging technology.

Taking a segment of MPJ & JAS tunnel structure as an example, use a Pundit 250 Array phased array ultrasonic imaging device to detect the concrete defects from the area 1 on the concrete free face and the area 2 on the steel plate free face (Fig. 35).

**Figure 36:**
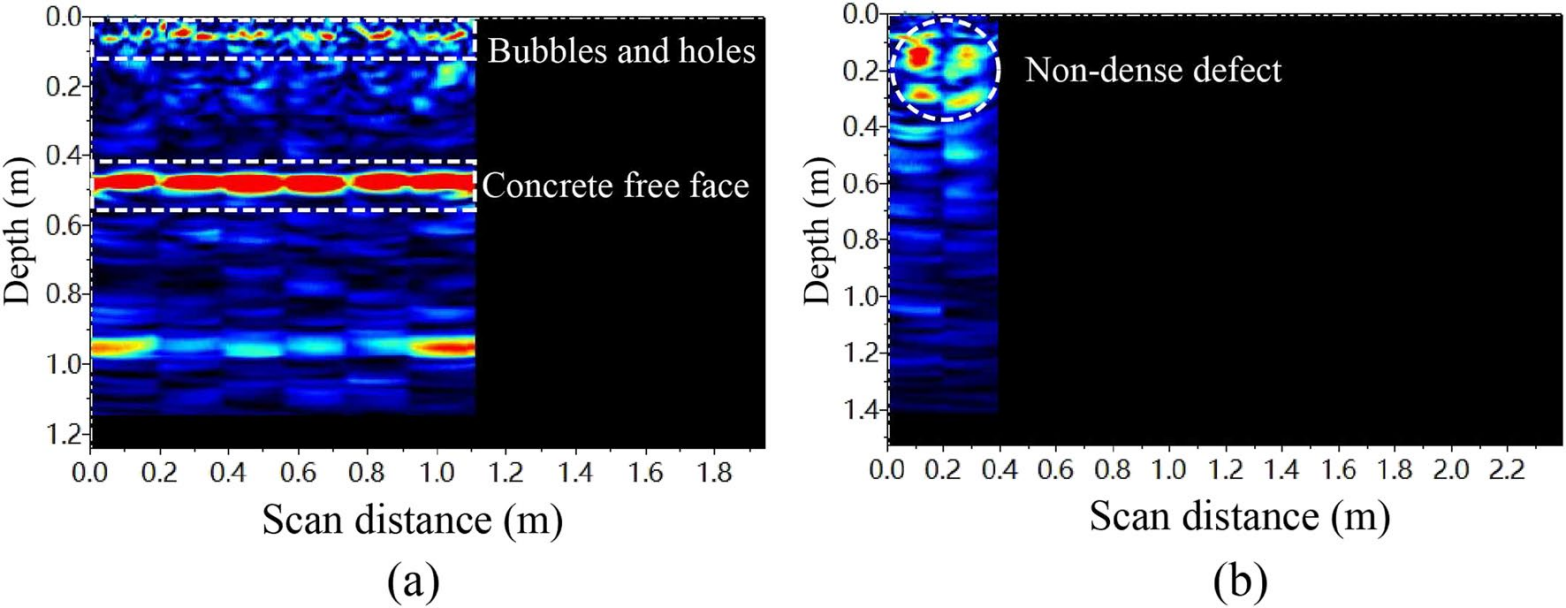
Concrete quality detection results.

The detection results of area 1 (Fig. 36a) show that there is a continuous red highlighted area at 0.5 m (equal to the segment thickness), which is the reflection signal generated by the signal of the phased array ultrasonic imager entering the air from the inner concrete, meaning that the concrete free face on the other side of the segment is detected. At 0.05 m below the surface, there are some small discontinuous red highlighted areas, indicating that there are some defects such as bubbles and holes on the concrete surface. However, there is almost no red highlighted area 0.05–0.5 m below the surface, indicating that the construction quality of inner concrete is quite good. In addition, the detection results of area 2 (Fig. 36b) show that the phased array ultrasonic imaging technology can penetrate the surface steel plate and detect the non-dense defect of the inner concrete at the depth of 0.1 –0.3 m. According to the above detection results, phased array ultrasonic imaging technology can effectively detect the concrete construction quality of MPJ & JAS tunnel.

## Conclusions

This paper proposed the MPJ & JAS method for constructing the tunnel undercrossing the existing road to solve the problem of more and more “dead end roads” in cities. The MPJ & JAS method assembles small cross-section elements to form a large cross-section tunnel, quite different from traditional methods. Therefore, the tensile test, bending test and FE analysis were conducted to verify and study the structure performance, a theoretical method was derived for design, and key construction equipment and technologies were developed. The following conclusions may be drawn:

1. MPJ & JAS method can significantly reduce the buried depth of the tunnel, the construction cost and time. It has high space utilization rate and the ability of flexible and variable cross-section setting.
2. The design of CT-shaped integrated joint, reference pipe, socket pipe and corner pipe and the development of high-performance grouting material effectively solved the MPJ & JAS tunnel connection. The tensile test showed that the CT-shaped integrated joint has excellent mechanical properties and can meet the bearing requirements of MPJ & JAS tunnel.
3. The bending test and FE analysis proved the reliability of the MPJ & JAS tunnel structure and found that the tensile failure of the CT-shaped integrated joint dominates the structural failure. Furtherly, the influence parameters study based on FE models indicated that steel plate thickness and filled concrete strength have great influence on structural performance, and the 16 mm thick steel plate and C50 concrete can guarantee both the bearing capacity and ductile failure. Although shear connectors have negligible influence on the ultimate load, it can be used as a constructional measure to reduce structural deformation.
4. Based on the failure behavior and reasonable assumptions, the theoretical design method considering the influence of the CT-shaped integrated joint could effectively predict the bending strength of the MPJ & JAS tunnel structure with an error of less than 10%.
5. The micro pipe jacking machine, soil reinforcement measure, hydraulic traction construction technology, high-precision guidance system and concrete construction quality detection method based on the phased array ultrasonic imaging technology were developed, effectively realizing the accurate and efficient construction of the MPJ & JAS tunnel.

## Data Availability

All data generated or analyzed during this study are included in this published article.

## Abbreviations

MPJ & JAS: Micro pipe jacking and joint assembly structure
HEP & JES: High-speed element pull and jointed element structure
RBJ: Roof-box jacking
PCR: Prestressed concrete roof
SCS: Steel–concrete–steel
URT: Under railway road tunneling
FE: Finite element
